# The importance of IgG glycosylation—What did we learn after analyzing over 100,000 individuals

**DOI:** 10.1111/imr.13407

**Published:** 2024-10-04

**Authors:** Jasminka Krištić, Gordan Lauc

**Affiliations:** ^1^ Genos Glycoscience Research Laboratory Zagreb Croatia; ^2^ Faculty of Pharmacy and Biochemistry University of Zagreb Zagreb Croatia

**Keywords:** ADCC, CDC, Fcγ receptors, glycosylation, IgG, IVIg

## Abstract

All four subclasses of immunoglobulin G (IgG) antibodies have glycan structures attached to the protein part of the IgG molecules. Glycans linked to the Fc portion of IgG are found in all IgG antibodies, while about one‐fifth of IgG antibodies in plasma also have glycans attached to the Fab portion of IgG. The IgG3 subclass is characterized by more complex glycosylation compared to other IgG subclasses. In this review, we discuss the significant influence that glycans exert on the structural and functional properties of IgG. We provide a comprehensive overview of how the composition of these glycans can affect IgG's effector functions by modulating its interactions with Fcγ receptors and other molecules such as the C1q component of complement, which in turn influence various immune responses triggered by IgG, including antibody‐dependent cell‐mediated cytotoxicity (ADCC) and complement‐dependent cytotoxicity (CDC). In addition, the importance of glycans for the efficacy of therapeutics like monoclonal antibodies and intravenous immunoglobulin (IVIg) therapy is discussed. Moreover, we offer insights into IgG glycosylation characteristics and roles derived from general population, disease‐specific, and interventional studies. These studies indicate that IgG glycans are important biomarkers and functional effectors in health and disease.

## 1 INTRODUCTION

Although the structure of all five classes of immunoglobulins is mostly made up of protein, all immunoglobulin classes also contain carbohydrate structures covalently attached to the protein backbone. Depending on the class, these carbohydrate structures contribute 2%–14% to the molecular weight of the entire immunoglobulin structure.^1^, ^2^, ^3^ These carbohydrate structures, known as glycans, are therefore an integral part of the immunoglobulin structure for all five immunoglobulin classes. This structural characteristic classifies immunoglobulins into a large and diverse group of proteins called glycoproteins. Immunoglobulins acquire glycans as co‐translational and post‐translational modifications during their synthesis and maturation in the ER and Golgi, in a process called glycosylation. Two types of glycans are found attached to protein part of immunoglobulins: N‐glycans and O‐glycans.^1^, ^2^, ^3^ N‐glycans are carbohydrate structures that attach to nitrogen atom of asparagine (Asn) amino acid in the protein.^4^ The attachment of N‐glycans is highly sequence‐specific, typically occurring in the consensus sequence Asn‐Xaa‐Ser/Thr, where Xaa can be any amino acid except proline. Another important structural component of immunoglobulins is O‐glycans, which are carbohydrates attached to the oxygen atom of serine (Ser) or threonine (Thr) residues within the protein.^5^ Unlike N‐glycans, O‐glycans do not attach to a known consensus sequence. Some immunoglobulin classes contain only N‐glycans (IgG [except IgG3 subclass], IgE, IgM), while others contain both N‐ and O‐glycans (IgG3, IgA1, and IgD).^1^, ^3^, ^6^

Among all classes of immunoglobulins, immunoglobulin G (IgG) undergoes the simplest glycosylation modification. Each IgG subclass (IgG1‐4), with the exception of IgG3, possesses a single highly conserved N‐glycosylation site where glycans attach specifically at position Asn 297 within constant regions 2 (CH2) of each of the two heavy chains.^3^, ^6^ In comparison, IgM has five glycosylation sites occupied by N‐glycans on each heavy chain, while IgE has as many as seven glycosylation sites on each heavy chain where N‐glycans can attach, one of which is not occupied by glycans.^1^, ^2^, ^3^

The relatively low complexity of glycosylation of IgG,^3^ combined with its higher abundance in human blood compared to other immunoglobulins (5 times more than IgA and over 10,000 times more than IgE),^7^, ^8^ which makes it more readily available for research—is factors that have contributed to IgG glycosylation being more thoroughly studied and characterized than that of other immunoglobulins.^3^, ^9^ This includes the variety of glycan structures attached to IgG, the functional roles of these glycans, the variability of IgG glycosylation within the general population, and the changes in IgG glycosylation associated with different diseases, physiological conditions, and in response to different interventions.^9^ Hence, this review will provide a synthesis of findings from extensive research on IgG glycosylation across diverse contexts: encompassing population‐based studies, disease‐specific investigations, interventional studies, and across different systems, primarily focusing on humans, while also presenting findings from studies involving mice and cell cultures.

## 2 GLYCOSYLATION SITES AND GLYCAN STRUCTURES PRESENT ON IgG ANTIBODIES

### 2.1 Glycan structures at the conserved Asn 297 N‐glycosylation site in the CH2 domain of the Fc portion

**FIGURE 1:**
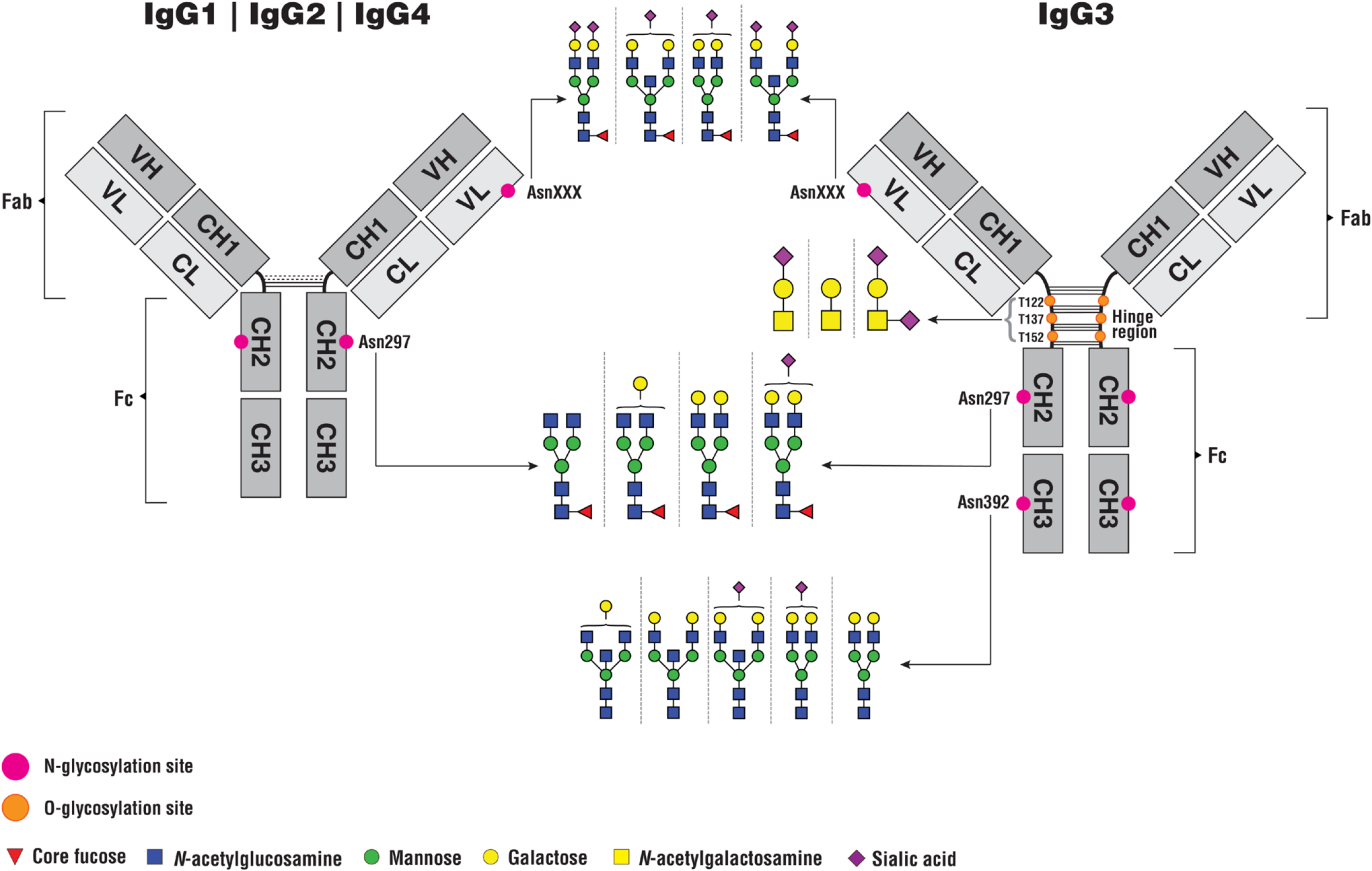
Glycosylation sites found on IgG1‐4 subclasses and the glycan structures present at these sites. Only the most abundant glycan structures found at each glycosylation site are shown.

As briefly mentioned earlier, three of the four IgG subclasses (IgG1, IgG2, and IgG4) each have only one conserved glycosylation site located at amino acid position Asn 297 in the CH2 constant region of the heavy chain within the Fc portion of IgG^3^, ^6^, ^10^ (Figure 1). This glycosylation site is occupied by N‐glycans. Since the Fc portion of IgG consists of two identical and symmetrical heavy chains, each IgG molecule typically carries two N‐glycans, accounting for 2%–3% of the molecular weight of IgG.^1^ The exception is subclass IgG3, which also contains the conserved Asn 297 N‐glycosylation site, but also has additional glycosylation sites^3^, ^6^, ^10^ (Figure 1), which will be described later in this review.

The glycosylation site at position Asn 297 in IgG can carry over 30 different N‐glycan structures.^11^ Therefore, microheterogeneity—the diversity of different glycan structures that can be attached to the same glycosylation site^12^—is relatively high for IgG.^11^, ^13^, ^14^ This means that individual IgG molecules in the body can have different glycan structures attached to their Fc portions.^11^, ^13^, ^14^ In other words, different glycoforms of IgG (each defined as a protein backbone with a specific glycan attached) can be found circulating through the body at the same time. Moreover, the pairing of glycans attached to each of the two heavy chains of an IgG molecule can be both homologous (same glycan structure on both heavy chains) and heterologous (different glycan structures on the two heavy chains).^15^ This further increases the number of possible IgG glycoforms, making the possible diversity of IgG glycoforms in the body quite extensive.

**FIGURE 2:**
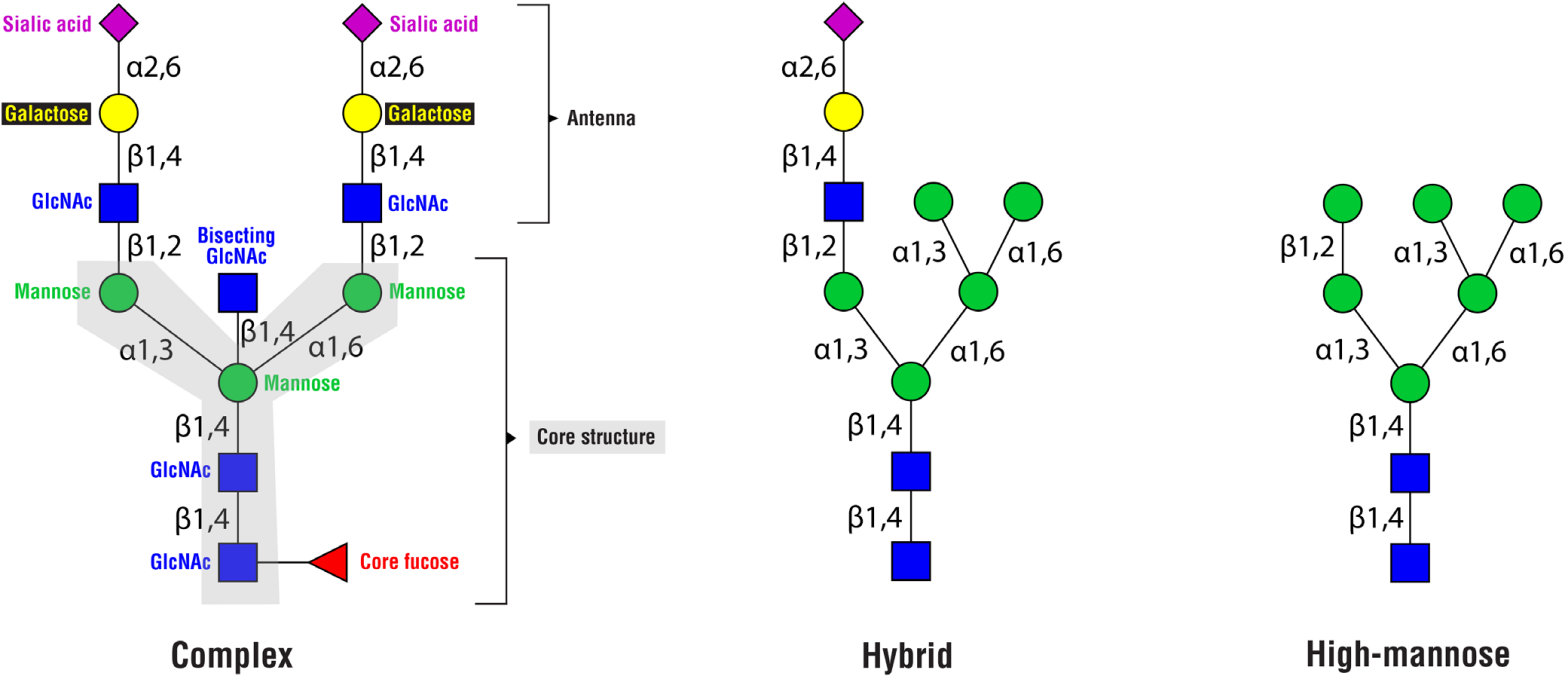
Three types of N‐glycans: Complex, hybrid, and high‐mannose. All three types of N‐glycans are present on human plasma IgG. Complex glycans are the most predominant, while hybrid and high‐mannose N‐glycans are present at very low abundance.

Regarding their structural properties, glycans attached to the Asn 297 position in the Fc portion of IgG are predominantly complex type glycans, although high‐mannose and hybrid types of N‐glycan structures are also present^11^ (Figure 2). However, both high‐mannose and hybrid glycans constitute less than 1% of the total glycans attached to IgGs present in the body.^11^, ^13^, ^16^ In contrast, glycans found on IgE and IgM include high‐mannose glycans at much higher abundance; moreover, both IgM and IgE contain glycosylation sites where high‐mannose type glycans predominantly bind.^3^, ^10^ All N‐glycans, including those attached to IgG, share the same pentasaccharide core structure^4^ (Figure 2). This core structure consists of two *N*‐acetylglucosamine (GlcNAc) residues, with the first one covalently attached to the Asn amino acid in the protein. Following these two residues in the core structure is a mannose residue, to which two additional mannose residues are attached as branches—one as the 3‐arm bound mannose and the other as the 6‐arm bound mannose. In complex types of N‐glycans, each of the two branched core mannose residues has further GlcNAc residues bound, to which galactose residues can attach. Additionally, sialic acid can bind to these galactose residues. Also, in complex N‐glycans, a fucose residue can attach to the first GlcNAc in the core structure, a modification known as core fucose. In complex glycans attached to IgG, the mannose residue in the core structure, to which two mannose residues are branched, can also have a GlcNAc residue bound, known as bisecting GlcNAc. Complex type glycans attached to IgG are mostly biantennary, meaning that the core structure branches into two antennae.^11^, ^13^, ^14^ Generally, each antenna in complex N‐glycans starts with a GlcNAc bound to two branched mannoses in the core structure. In the case of IgG glycans, one GlcNAc‐initiated antenna is bound to the 6‐arm bound mannose and the other to the 3‐arm bound mannose. In contrast to complex N‐glycans, hybrid N‐glycans have only one of the two branched mannose core residue—specifically the 3‐arm bound—further elongated in the antenna by the attachment of a GlcNAc residue, while to the 6‐arm bound mannose, one or more mannose residues are attached^4^ (Figure 2). On the other hand, high‐mannose N‐glycans, as their name implies, have only additional mannose residues bound to the core structure (Figure 2), without attachment of GlcNAc to either of the two branched core mannose residues.^4^

The predominant type of glycans attached to the Asn 297 site in the Fc portion of IgG is complex glycans^11^, ^13^ (Figure 1). However, while the variety of complex N‐glycan structures found in nature is extensive,^4^ only a fraction of these complex glycans is found attached to IgG, with some being more common on IgG and others rare.^11^, ^13^, ^14^ For instance, as mentioned earlier, the majority of complex glycans attached to IgG are biantennary types of complex N‐glycans, whereas triantennary glycans found attached to IgA are absent on IgG.^17^, ^18^, ^19^ On average, about 35% of the total N‐glycans found on the Fc portion of IgGs in human blood are biantennary complex glycans with GlcNAc residues attached to the core structure, with these GlcNAcs not further modified by galactose (known as agalactosylated glycans).^20^ N‐glycans with galactose account for around 65% of all glycans found on position Asn 297 of circulating IgGs.^11^, ^20^ Within this 65%, almost 40% are glycans with galactose bound to only one of the two possible GlcNAc‐initiated antennae (monogalactosylated glycans), 15% correspond to glycans with galactoses attached to both antennae (digalactosylated glycans), and approximately 12% correspond to glycans that have sialic acid bound to galactose on one of the antennae (monosialylated glycans).^11^, ^20^ Over 90% of all glycans attached to Fc portion of IgGs in human blood contain core fucose (core‐fucosylated glycans).^11^ Furthermore, approximately 10%–15% of the total IgG Fc glycans contain bisecting GlcNAc.^11^, ^14^, ^20^ Additionally, differences in Fc glycosylation between IgG subclasses have been reported, with the IgG2 subclass showing higher levels of glycans with core fucose and glycans lacking galactose, but lower levels of glycans with one or two galactoses, as well as glycans with bisecting GlcNAc, compared to IgG1 and IgG4 subclasses.^21^, ^22^ However, data regarding the levels of glycans with sialic acid among IgG subclasses remain contradictory.

### 2.2 Glycan structures at the N‐glycosylation sites in the variable region of the Fab portion

In addition to the conserved N‐glycosylation site at Asn 297 in the Fc portion of IgG, it has been demonstrated that 15%–25% of circulating IgG antibodies also contain N‐glycosylation sites in the variable region of the Fab portion (Figure 1).^11^, ^23^, ^24^ These glycosylation sites, which are not present in all IgG molecules, are acquired during somatic hypermutation.^25^ Although the acquisition of Fab N‐glycosylation sites on IgG molecules and their location within the variable region are random processes, research indicates that a certain selection pressure for Fab N‐glycosylation site acquisition during the affinity maturation of B cells exists.^25^ This is evidenced by the fact that IgG4 has elevated levels of N‐glycosylation sites compared to other IgG subclasses, similar to the levels observed in the IgE immunoglobulin class.^25^, ^26^ This could be connected to Th2 immune responses, which promote the production of both IgE and IgG4.^25^ Additionally, it has been observed that certain antigen‐specific IgG antibodies have increased levels of Fab glycans compared to total circulating IgG. Specifically, in the case of IgG anticitrullinated protein antibodies (ACPA), Fab glycans are present on over 90% of ACPA IgGs.^27^, ^28^ These Fab glycans, which appear to arise from the non‐random accumulation of N‐glycosylation sites in the variable region of ACPA IgG, are thought to contribute to the selection of autoreactive B cells and the development of autoimmunity in rheumatoid arthritis (RA).^29^

A difference has been observed in the structural characteristics of glycans that are more commonly found attached to the Fab portion of IgG compared to those more abundantly attached to the Fc portion.^11^ Among glycans found in the variable region of the Fab portion of IgG, there is a higher abundance of glycans with bisecting GlcNAc and higher levels of glycans with sialic acid, with a substantial proportion corresponding to those carrying two sialic acids, which are almost completely absent among Fc glycans (present with an abundance of less than 1%). On the other hand, among glycans attached to the Fab portion, there is a lower level of core‐fucosylated glycans compared to Fc IgG glycans. Moreover, most Fab glycans contain galactose on at least one branch, while glycans without galactose are rare among Fab glycans. Additionally, high‐mannose type glycans are found among Fab glycans at an abundance of approximately 4%, which is greater than the abundance observed for the same type of glycans in the Fc portion.^11^

### 2.3 Glycan structures at the Asn 392 N‐glycosylation site in the CH3 domain of the Fc portion of the IgG3 subclass

Regarding the glycosylation of the IgG3 subclass, this subclass contains some additional glycosylation sites not found in other IgG subclasses^3^, ^6^ (Figure 1). In addition to the N‐glycosylation site Asn 297 that is present in all four IgG subclasses in the CH2 domain of the Fc portion, several allotypes of IgG3 subclass contain an extra N‐glycosylation site—Asn 392, located in the CH3 domain of the Fc portion.^6^, ^30^ This glycosylation site is also found in one IgG2 allotype,^6^ although not much is known about its glycosylation, as it is not as well characterized as in the IgG3 subclass. Contrary to the Asn 297 glycosylation site, which is practically 100% occupied by glycans, the additional Asn 392 glycosylation site in IgG3 has a reported N‐glycan site occupancy of around 12%. The glycan structures that mainly attach to this Asn 392 N‐glycosylation site in IgG3 subclass, differ from those glycans that attach to the Asn 297 N‐glycosylation site conserved among all IgG subclasses. Although both Asn 392 and Asn 297 N‐glycosylation sites predominantly carry biantennary complex types of N‐glycans, the glycans found on the Asn 392 N‐glycosylation site in the IgG3 subclass mostly lack core fucose, whereas those on Asn 297 mostly contain core fucose. Additionally, N‐glycans commonly found on the Asn 392 site contain bisecting GlcNAc. Regarding other characteristics of the glycans at this site, it is observed that they may lack galactose, have one or two galactose residues, or also contain sialic acid, similar to the glycans found at the Asn 297 site. Furthermore, a high‐mannose type of glycan, which contains five mannose residues, is found on the Asn 392 site with an abundance of around 9%.^30^

### 2.4 Glycan structure at the O‐glycosylation sites in the hinge region of the IgG3 subclass

The hinge region of the IgG3 subclass, which connects the Fab and Fc portions of IgG, is approximately four times longer than the hinge region of other IgG subclasses and contains three glycosylation sites reported to be occupied by O‐glycans^6^, ^31^, ^32^ (Figure 1). No O‐glycans are reported for other IgG subclasses.^3^ The glycan occupancy of each of these three IgG3 hinge O‐glycosylation sites (Thr 122, Thr 137, and Thr 152) was determined to be around 10%.^31^ The O‐glycan structures that bind to these sites are mostly core 1 O‐glycans, a highly abundant type of O‐glycan found on many glycoproteins. The structure of core 1 O‐glycans consists of an *N*‐acetylgalactosamine (GalNAc) residue bound to a threonine or serine amino acid in the protein backbone, with a galactose residue bound to protein‐bound GalNAc in a β1‐3 linkage (Galβ1‐3GalNAc‐Ser/Thr).^5^ On IgG3, these core 1 O‐glycans are without sialic acid, or may carry one or two sialic acid residues.^31^

## 3 EFFECTS OF GLYCOSYLATION ON IgG STRUCTURE

Glycans attached to IgG are not just decorative additions to the protein backbone or merely contributors to the overall molecular weight of the IgG molecule; research has demonstrated that they significantly influence the conformation of the IgG molecule. This structural impact has direct implications for the interaction of IgG with Fc gamma (Fcγ) receptors and other interacting molecules, such as the complement component C1q, through which IgG antibodies exert their effector functions.^33^

### 3.1 Effect of glycan size on IgG structure

The examination of the crystal structure of the IgG Fc fragment carrying Asn 297 N‐glycans with varying numbers of monosaccharide residues, ranging from processed and larger N‐glycan structures with 10 monosaccharide residues, including two terminal galactoses, to very simple N‐glycans with only the core structure or even more truncated N‐glycan structures (with only 6 or 4 monosaccharide residues), showed that the distance between the two CH2 domains is larger when larger N‐glycans are attached to the Fc portion.^34^ Conversely, when simpler glycans with fewer monosaccharides are attached, the CH2 domains are positioned closer together.^34^ This demonstrates that the degree of openness of the Fc portion's conformation depends on the specific N‐glycans that are attached.

### 3.2 Effect of the presence versus absence of glycans on IgG structure

Studies comparing the crystal and solution structures of glycosylated Fc fragments with those completely devoid of glycans (aglycosylated/deglycosylated Fc fragments), as well as the solution structure of complete IgG (containing both the Fab and Fc portions, undigested into fragments) with and without glycans, showed that glycans play a role in stabilizing the conformation of the CH2 region of IgG, especially within the subregion encompassing the CE loop.^33^, ^35^, ^36^ This stabilization supports maintaining an open conformation of the Fc portion of IgG and ensures that the protein and glycan parts of the IgG molecule, which are essential for establishing interaction with Fcγ receptors, are exposed and properly oriented.^33^, ^35^, ^36^ On the other hand, it has been shown that the Fc portion of IgG lacking glycans (observed based on the structure of both the digested Fc fragment only and the total IgG structure) is structurally more flexible and can adopt a range of different conformational states, from more closed to more open.^35^, ^36^ This unstable and dynamic structure of non‐glycosylated IgG is likely the reason behind the widespread observation of its abrogated interaction with Fcγ receptors.

### 3.3 Effect of glycan composition of IgG structure

While glycans typically promote an open conformation of the IgG Fc portion, indications suggest that their influence on IgG structure can be highly complex, depending on the glycan composition, with profound effects on downstream immune responses. Several studies have reported that IgG glycans containing galactose confer a significant stabilizing effect on the structure of the CH2 domain of IgG.^37^, ^38^, ^39^, ^40^ Research has also shown that glycans with sialic acid attached at position Asn 297 induce a significant structural change in the CH2 region.^41^ This change causes the Fc portion of IgG to adopt a more closed conformation compared to IgG with glycans lacking sialic acid. The closed conformation of IgG carrying sialylated glycans is distinct from that observed in IgG completely lacking glycans. Furthermore, it has been demonstrated that this specific structural conformation enables sialylated IgG to interact with the C‐type lectin DC‐SIGN, thereby initiating an anti‐inflammatory immune response.^41^ The degree to which glycan composition influences IgG structure is still in question, especially since later research did not corroborate the initially reported effects of sialic acid on inducing IgG conformational change.^42^

### 3.4 Effect of IgG glycosylation on the thermal stability of IgG

It should also be mentioned that IgG without glycans and IgG with simple glycans (limited to the core N‐glycan structure with core fucose) has shown reduced thermal stability.^41^, ^43^ On the other hand, IgG with galactose‐containing glycans is reported to be more thermally stable than other glycosylated IgG variants,^44^ although the impact of galactose on thermal stability has not been consistently confirmed.^43^

## 4 EFFECTS OF GLYCOSYLATION ON EFFECTOR FUNCTIONS OF IgG

IgG is involved in various immune responses, including antibody‐dependent cellular phagocytosis (ADCP), antibody‐dependent cell‐mediated cytotoxicity (ADCC), complement‐dependent cytotoxicity (CDC), induction of cytokine production and other inflammatory mediators, and anti‐inflammatory immune responses.^45^ To initiate these immune responses, IgG interacts with Fcγ receptors (FcγR) present on various types of immune cells, the C1q component of complement, as well as other cell‐bound or soluble molecules such as DC‐SIGN, CD22, CD23, and TRIM21.^32^, ^46^ These interactions between IgG and receptors, complement components, and other molecules are crucial for activating the downstream signaling pathways necessary for carrying out diverse immune responses.^46^, ^47^

### 4.1 Effect of the complete absence of IgG glycosylation on IgG effector functions

Numerous studies have demonstrated that IgG lacking N‐glycans, whether through enzymatic deglycosylation, production in cell lines to generate agalactosylated IgG, or specific removal of N‐glycans at the conserved Asn 297 glycosylation site, as well as using only the digested Fc fragment without N‐glycans, exhibits significantly reduced or completely abolished binding to Fcγ receptors and the complement component C1q.^33^, ^43^, ^48^, ^49^, ^50^, ^51^, ^52^, ^53^, ^54^ These studies also highlighted the crucial role of N‐glycans attached to the Asn 297 site in the Fc portion of IgG for these interactions.^33^, ^43^, ^53^ More specifically, IgG lacking glycans has approximately a 40‐fold reduced affinity for binding the high‐affinity activating receptor FcγRI, which can bind IgG in the form of monomers as well as multimeric IgG immune complexes (IgG ICs).^43^, ^53^ This reduction in affinity is noted for monomeric IgG lacking glycans, whereas it appears that IgG in the form of ICs maintains a high binding affinity for FcγRI regardless of glycan removal.^52^ Regarding binding to low‐affinity FcγRs—both the activating FcγRIIa/c and FcγRIIIa/b, as well as the inhibitory FcγRIIb, which primarily bind IgG in the form of immune complexes (IgG ICs)—studies have shown that IgG lacking glycans either fails to bind to these receptors or exhibits drastically reduced binding.^33^, ^43^ However, it seems that the binding between deglycosylated IgG and low‐affinity receptors can be somewhat enhanced by increasing the size of the IgG ICs, although this improvement is highly dependent on the IgG subclass and the type of receptor.^52^ Furthermore, it appears that by introducing certain mutations in the IgG Fc portion, it is possible to produce aglycosylated IgG that retains binding similar to or even higher than glycosylated IgG to some low‐affinity receptors, while also exhibiting only a 10‐fold reduction in binding affinity for the high‐affinity receptor FcγRI.^55^

Since the interaction between the Fc portion of IgG and FcγR is crucial for triggering various immune effector responses such as ADCC and phagocytosis, studies have shown that reduced or absent binding of glycan‐deficient IgG to FcγRs directly translates into a substantially reduced or absent ability of such IgG to induce ADCC and ineffective depletion of target cells.^48^, ^54^

Research has also shown that IgG antibodies without glycans have reduced binding to complement component C1q, which significantly impairs their capacity to activate C1q‐dependent CDC.^48^, ^49^, ^54^

### 4.2 Effect of partially glycosylated (hemiglycosylated) IgG on its effector functions

A study comparing the binding of completely glycosylated (both heavy chains of the Fc portion of IgG having glycans) and hemiglycosylated (where only one heavy chain of the Fc portion of IgG has glycans while the other does not) recombinantly produced monoclonal IgG1 antibodies with FcγRs and the C1q component of complement revealed that hemiglycosylated IgG showed a 2–3‐fold decrease in binding affinity to all FcγRs, resulting in a 3.5‐fold lower ADCC activity.^56^ Additionally, hemiglycosylated IgG1 exhibited a 20% reduction in binding to C1q compared to fully glycosylated IgG1.^56^ These findings underscore the importance of proper glycosylation of IgG, with both conserved sites occupied by glycans, for ensuring fully functional IgG antibodies capable of effectively interacting with receptors and other molecules to elicit various effector functions.

### 4.3 Minimal glycosylation required for IgG to exhibit effector functions

As discussed above, IgG that lacks glycans cannot form functionally relevant interactions with Fcγ receptors, except for FcγRI, which is a high‐affinity receptor for IgG and retains a higher level of residual binding with glycan‐deficient IgG compared to low‐affinity FcγRs.^43^, ^52^ However, research has demonstrated that IgG with just one monosaccharide attached to Asn 297 position in Fc portion is sufficient to enable interactions with FcγR that can direct IgG effector functions.^54^ In a study using IgG1 and IgG3 switch variants of the therapeutic human monoclonal anti‐CD20 antibody rituximab, which had only one GalNAc residue or fucosylated GalNAc (two monosaccharides) attached to the Asn297 site (produced by EndoS enzyme digestion), it was shown that B cells could be depleted as effectively as with fully glycosylated rituximab (with larger glycans attached).^54^ In contrast, agalactosylated rituximab lacking this single GalNAc monosaccharide lost the ability to efficiently deplete B cells.^54^ When considering binding affinity, minimally glycosylated IgG (with 1–2 monosaccharides) appears to have reduced binding to FcγRs compared to IgG with larger glycans.^44^, ^52^ This is illustrated, for example, by the 10‐fold lower FcγRIIIa affinity of IgG1 with just one GalNAc residue compared to IgG1 with a 10‐monosaccharide glycan.^33^ Nevertheless, despite this reduction in binding affinity, it appears that minimally glycosylated IgG is still capable of effectively activating FcγRs and triggering downstream effector functions.^54^ Furthermore, it has been shown that binding of IgG to FcγRs is influenced by the size and composition of the attached glycans. IgG with only a fucosylated core glycan (pentasaccharide core + core fucose), as well as IgG carrying octa‐ or nonasaccharide glycans with core fucose and two GalNAcs, with or without one additional galactose attached to the core structure, exhibit reduced binding to FcγRs (all except FcγRI), compared to IgG with a decasaccharide glycans with two galactoses, which showed improved binding.^43^

While already minimal glycosylation of IgG appears sufficient to activate FcγRs, human IgG1 and IgG3 antibodies with only one monosaccharide (GalNAc) or two monosaccharides (GalNAc and core fucose) showed reduced binding to the C1q component of the complement system, similar to IgG lacking glycans entirely.^44^, ^54^ As a result, it has been demonstrated that minimally glycosylated IgG antibodies are unable to activate CDC.^54^

Of note, some studies have shown promise in using the EndoS enzyme, which specifically cleaves Fc IgG glycans, producing minimally glycosylated IgG with only disaccharides attached to the Fc portion (GalNAc plus core fucose), for alleviating inflammation in certain autoimmune disease.^57^, ^58^, ^59^ These studies indicate that while in some cases, minimally glycosylated IgG (carrying only a disaccharide glycan) can activate immune effector responses,^54^ in other cases, it can disrupt interaction of IgG with receptors and the activation of downstream effector functions, such as pro‐inflammatory responses driving autoimmune diseases.^54^, ^57^, ^59^ In addition to affecting the interaction with FcγRs as a mechanism through which minimally glycosylated IgG suppresses inflammation, another explanation for suppressed inflammation could be prevention of complement‐mediated pro‐inflammatory immune responses, since it has been shown that minimally glycosylated IgG does not establish functional interactions with complement components.^44^, ^54^ Furthermore, it has also been demonstrated that minimally glycosylated IgG (after treatment with EndoS enzyme) can decrease the formation of immune complexes (ICs) by affecting Fc‐Fc interactions, presenting a strategy for treating diseases with IC‐mediated inflammation like arthritis.^58^ There are speculations that other potential mechanisms through which minimal Fc glycosylation of IgG could reduce inflammation are possible but not yet fully understood.^57^, ^59^ Overall, it appears that the influence of minimally glycosylated IgG on the activation or suppression of different effector functions is highly dependent on the IgG subclass, the specific disease context, and the overall immune system environment, considering all involved components (including FcγRs, complement, and IC formation).^54^, ^57^, ^58^, ^59^

## 5 MODULATION OF IgG EFFECTOR FUNCTIONS BY DIFFERENTIAL GLYCOSYLATION AT THE CONSERVED ASN 297 FC N‐GLYCOSYLATION SITE

**FIGURE 3:**
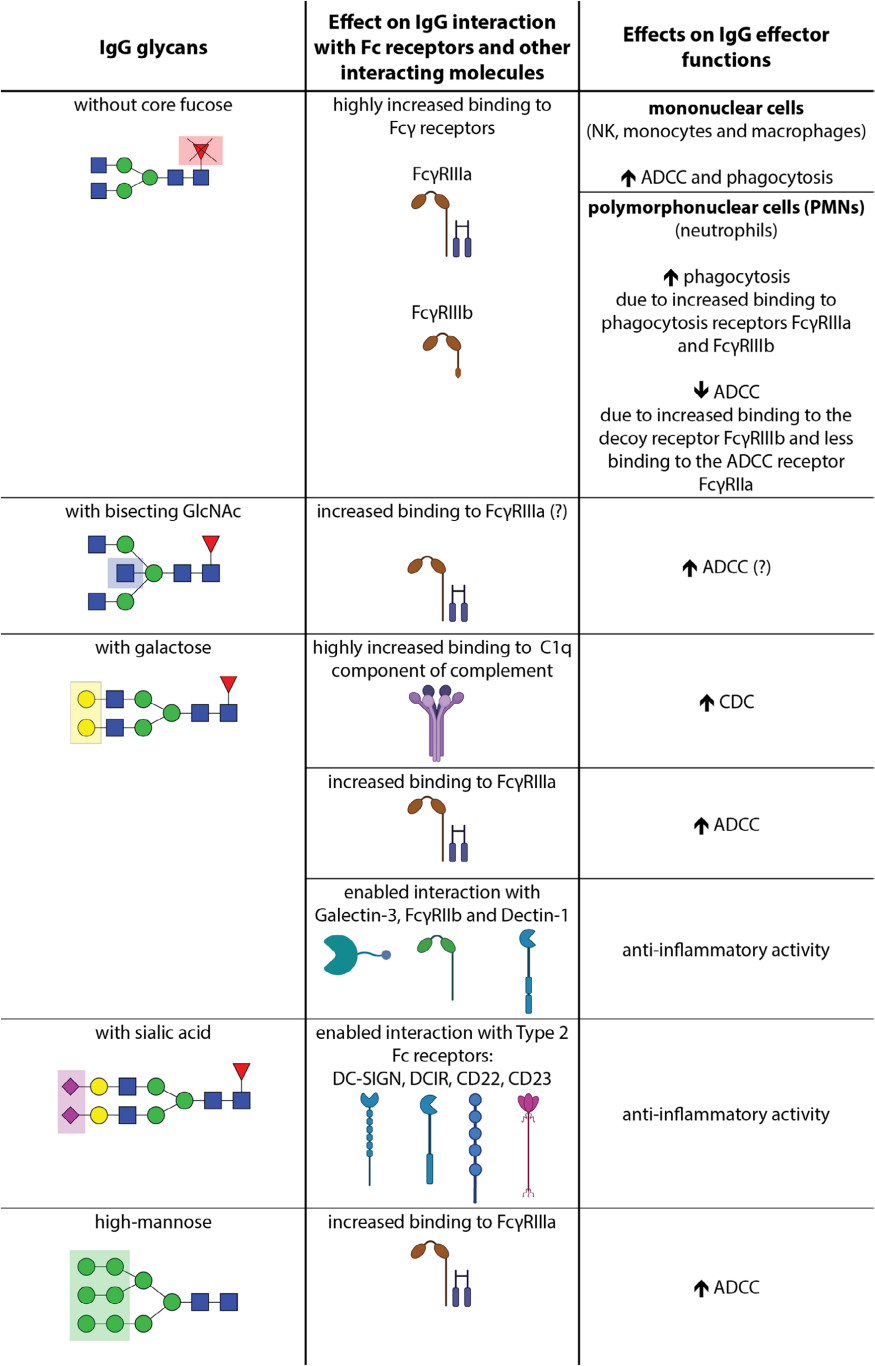
Effects of differential Fc glycosylation on IgG effector functions. The icons used in the creation of this figure were sourced from BioRender.com, and glycan structures were created using GlycoWorkbench. The (?) symbol denotes uncertainty based on current literature.

It has been well documented that the composition of glycan attached to the conserved 297 N‐glycosylation site in the Fc portion of IgG antibodies greatly influences IgG effector functions (Figure 3). Certain glycan structures can enhance specific IgG effector function, preferentially activate some effector functions, and inhibit others. Therefore, it has been recognized that glycosylation of IgG acts as an important switch between pro‐inflammatory and anti‐inflammatory immune responses.^6^, ^32^, ^46^, ^60^, ^61^

### 5.1 Effect of IgG glycans without core fucose on IgG effector functions

That the effector function of IgG can be strongly affected by the composition of glycans attached to it is best evidenced by the effect of the presence versus absence of core fucose in IgG glycans on ADCC function. A large body of research has demonstrated that IgG antibodies whose Asn 297 glycans do not contain core fucose have significantly increased affinity for both FcγRIIIa and FcγRIIIb receptors (from 10‐ to 50‐fold increased affinity, depending on the study) compared with IgG with core‐fucosylated glycans^62^, ^63^, ^64^, ^65^, ^66^, ^67^, ^68^, ^69^ (Figure 3). The absence of core fucose on IgG glycans does not affect IgG affinity for other Fcγ receptors, nor does it affect the interaction with the C1q component of complement and CDC activation,^44^, ^64^, ^68^, ^69^ although some studies have reported slightly improved affinity for FcγRIIb, FcγRIIc, and the Arg131 polymorphic form of FcγRIIa receptors.^62^, ^67^ Research has revealed that both nonfucosylated glycans on IgG and the glycan at the Asn 162 position in FcγRIII are responsible for this increased binding affinity.^70^ Some studies indicate the importance of glycan‐glycan interactions between glycans on IgG and the FcγRIIIa receptor,^71^, ^72^ while another study suggests that the increased affinity arises from conformational adjustments.^73^

The FcγRIII receptors are expressed on many immune effector cells—FcγRIIIa primarily on natural killer (NK) cells, but also on monocytes and macrophages, and FcγRIIIb exclusively on neutrophils.^74^, ^75^ It has been shown that the increased binding affinity between IgG lacking core fucose and the FcγRIIIa receptor leads to significantly enhanced ADCC by NK cells.^62^, ^68^, ^76^, ^77^ (Figure 3). Additionally, some studies have shown that non‐core‐fucosylated IgG improves ADCC and phagocytosis mediated by other FcγRIIIa‐expressing mononuclear cells, such as monocytes and macrophages.^62^, ^64^, ^65^, ^66^, ^78^ However, the way in which the presence or absence of core fucose on IgG influences the effector functions of polymorphonuclear cells (PMNs) is more complex (Figure 3). There is research showing that non‐core‐fucosylated IgG enhances phagocytosis by PMN neutrophils through increased binding to FcγRIIIb expressed on these cells.^79^ Another study, however, demonstrated that IgG‐induced phagocytosis in PMN neutrophils is mediated not by FcγRIIIb but by the FcγRIIIa receptor, which is also present on neutrophils but at lower levels.^80^ Furthermore, it has been shown that ADCC by PMN neutrophils is not enhanced in the presence of IgG lacking core fucose but is decreased.^81^, ^82^ The explanation for this seems to lie in the observation that the primary receptor on neutrophils mediating ADCC activation by IgG is the FcγRIIa receptor and not the FcγRIIIb receptor, which shares a high level of similarity with the FcγRIIIa receptor, the primary receptor for activation of NK cell‐mediated ADCC.^82^ Research indicates that in the presence of non‐core‐fucosylated IgGs, which have an enhanced binding affinity for FcγRIIIb, these IgGs will bind more readily to this receptor on neutrophils. As a result, fewer IgGs will be available to bind to FcγRIIa on neutrophils, which is essential for the activation of neutrophil‐mediated ADCC. Thus, FcγRIIIb seems to function as a decoy receptor on neutrophils for non‐core‐fucosylated IgG, resulting in reduced ADCC by neutrophils when non‐core‐fucosylated IgG is present.^82^ This aligns with findings that IgG antibodies with high core fucosylation induce more efficient ADCC by neutrophils compared to those with low core fucosylation.^81^

It should also be mentioned that research has demonstrated that the enhancement of ADCC by non‐core‐fucosylated IgG compared to core‐fucosylated IgG is particularly pronounced at lower IgG concentrations, from 0.01 to 1 μg/mL.^62^, ^83^ Furthermore, it has been shown that IgG antibodies of all subclasses (IgG1, IgG2, IgG3, and IgG4) lacking core fucose in their glycans exhibit increased binding to FcγRIIIa and FcγRIIIb compared to core‐fucosylated IgGs of all subclasses, although the extent of this increase depends on the IgG subclass.^64^, ^69^ However, findings regarding ADCC enhancement depending on the IgG subclass are mixed. One study reported that non‐core‐fucosylated variants of all IgG subclasses enhance ADCC function mediated by IgG‐interacting effector cells.^64^ In contrast, another study observed increased ADCC only for non‐core‐fucosylated IgG1 and IgG3, while IgG2 and IgG4 antibodies without core fucose did not enhance ADCC.^69^

It has also been shown in several studies that IgG antibodies lacking core fucose and containing galactose in their glycans exhibit even greater binding to FcγRIIIa and enhanced ADCC compared to IgG antibodies that only lack core fucose.^43^, ^44^, ^67^, ^68^ However, it has been demonstrated that the effect of lacking fucose on improving IgG affinity for FcγRIIIa and enhancing ADCC is greater than the effect of the presence of galactose. Additionally, one study examined how three different types of N‐glycans that are found attached to IgG affect the affinity of IgG for the FcγRIIIa receptor and the ADCC response.^65^ These types include the complex N‐glycans, which are abundantly found on IgG, as well as the less commonly found hybrid and high‐mannose N‐glycans on IgG. This study demonstrated that IgG with non‐core‐fucosylated forms of both complex and hybrid glycans had significantly increased affinity for FcγRIIIa and exhibited enhanced ADCC compared to IgG with core‐fucosylated forms of these glycans, while having similarly high FcγRIIIa affinity and ADCC responses as IgG with high‐mannose glycans.^44^, ^65^

It is also relevant to point out how the absence versus presence of core fucose on IgG affects binding to two different polymorphic variants of FcγRIIIa, which differ in the amino acid present at position 158: one variant has phenylalanine (Phe), while the other has valine (Val) at this position. It is known that IgG antibodies with high levels of core fucosylation have approximately five times higher affinity to the FcγRIIIa Val 158 variant compared to the FcγRIIIa Phe158 variant, and therefore, the FcγRIIIa Val 158 variant is referred to as the high‐affinity variant of the FcγRIIIa receptor, while the FcγRIIIa Phe 158 variant is known as the low‐affinity variant.^69^, ^76^ Additionally, in the presence of core‐fucosylated IgG, NK cells expressing the FcγRIIIa Val 158 variant have been shown to be approximately three times more potent in ADCC than NK cells expressing the FcγRIIIa Phe 158 variant.^76^ On the other hand, IgGs with low core fucosylation exhibit similar binding affinity for both FcγRIIIa polymorphic variants (with only a slightly higher affinity for the FcγRIIIa Val 158 variant), but this affinity is greater for both FcγRIIIa receptor variants compared to core‐fucosylated IgG antibodies.^69^ It has also been shown that IgG with low core fucosylation induces similarly efficient ADCC responses through both FcγRIIIa receptor variants, which are significantly higher compared to the ADCC responses induced by highly core‐fucosylated IgG.^76^

This proven ability of IgG without core fucose to enhance ADCC has been widely utilized to enhance the efficacy of therapeutic monoclonal antibodies that rely on ADCC to treat various diseases.^84^ Many studies have demonstrated that non‐core‐fucosylated forms of certain therapeutic antibodies induce more potent ADCC against cancer cells, viruses, and cells driving inflammation and autoimmunity compared to fucosylated forms of these antibodies.^84^, ^85^, ^86^ Notably, several non‐core‐fucosylated therapeutic antibodies are already approved for clinical use (for treating lymphomas and asthma), and many others are in clinical trials or development for the treatment of various types of cancers, viral infections, and inflammatory and autoimmune diseases.^84^ Although numerous studies have demonstrated the high potential of non‐core‐fucosylated therapeutic antibodies for improving the treatment of various diseases, several observations have identified factors that may limit the therapeutic potential of these antibodies in real physiological conditions. These observations include, but are not limited to, the finding that endogenous IgG in human plasma can reduce the ADCC response induced by therapeutic antibodies.^66^, ^83^ Additionally, the ability of non‐core‐fucosylated therapeutic antibodies to enhance ADCC (or phagocytosis) is influenced by the type of effector cells involved in the response,^69^ as evidenced by the differing effects of therapeutic antibodies with low core fucosylation on ADCC mediated by mononuclear cells versus polymorphonuclear (PMN) cells.^81^

### 5.2 Effect of IgG glycans with bisecting GlcNAc on IgG effector functions

Several studies have reported that IgG preparations containing higher levels of glycans with bisecting GlcNAc showed increased binding to FcγRIIIa and led to increased ADCC activity^78^, ^87^, ^88^, ^89^, ^90^, ^91^ (Figure 3). However, in some of these studies, the glycan structures identified as responsible for increased FcγRIIIa binding and enhanced ADCC not only contained bisecting GlcNAc but also lacked core fucose.^87^, ^90^ This is not surprising, as studies have shown that the addition of bisecting GlcNAc restricts the addition of core fucose by the corresponding fucosyltransferase enzyme, likely due to steric effects.^92^, ^93^ Given that the absence of core fucose is well‐documented to significantly increase IgG binding to FcγRIIIa and enhance ADCC,^84^ based on these studies it is difficult to conclude whether the observed improvement of FcγRIIIa binding and ADCC is due solely to the absence of core fucose or if bisecting GlcNAc also has an effect. Few studies, where the effect of core fucose was more carefully controlled to isolate the impact of bisecting GlcNAc on FcγRIIIa binding and ADCC, demonstrated that highly core‐fucosylated IgG preparations with high levels of glycans containing bisecting GlcNAc (>70%) exhibited enhanced ADCC (7–10 fold) and increased FcγRIIIa binding compared to preparations without bisecting GlcNAc.^78^, ^88^, ^89^ On the other hand, the effect of bisecting GlcNAc on FcγRIIIa binding and ADCC in IgG antibodies with low levels of core fucosylation remains unclear. However, available studies suggest that while the absence of core fucose greatly enhances IgG binding to FcγRIIIa and ADCC activity,^78^ the presence of bisecting GlcNAc might provide a small additional improvement.^91^ No effect, or only a minimal effect, on CDC, depending on the presence or absence of bisecting GlcNAc in IgG glycans has been observed so far.^68^, ^89^

### 5.3 Effect of IgG glycans with and without galactose on IgG effector functions

Many studies have shown that IgG antibodies with galactose in their Fc glycans have higher binding affinities for the C1q component of the classical complement pathway, which has also been associated with enhanced complement cascade activation and CDC^44^, ^89^, ^94^, ^95^, ^96^ (Figure 3). The impact of the presence of galactose in IgG glycans on IgG activity, enhancing C1q binding and subsequent CDC, has only been seen in the IgG1 and IgG3 subclasses. The IgG2 and IgG4 subclasses exhibit poor C1q binding and CDC activity, and it seems that even with the addition of galactose to their Fc glycans, these subclasses remain less potent in initiating complement activation.^64^, ^95^, ^97^ It has also been demonstrated that the presence of galactose in IgG glycans enhances IgG binding affinity for the FcγRIIIa receptor, which subsequently enhances ADCC activity of FcγRIIIa‐expressing effector cells, independently of whether core fucose is present^43^, ^44^, ^67^, ^68^, ^98^ (Figure 3). However, as mentioned before, the absence of core fucose in IgG glycans has a much larger impact on increasing IgG binding to FcγRIIIa and downstream ADCC activation than the presence of galactose. There are also studies that reported that IgG antibodies with two galactoses in their glycans exhibit increased binding affinity not only for the FcγRIIIa receptor but also for the FcγRIIIb and FcγRIIa/b receptors, compared to IgG antibodies that either lack galactose or contain only one galactose.^43^, ^67^

In the context of research on how IgG glycans with galactose affect complement activation, it is important to mention further research into IgG activation of complement that has revealed that IgG antibodies form hexameric complexes on the cell surface.^99^ This research has shown that hexameric complexes enhance binding of IgG to C1q component, which has six antibody‐binding globular domains, leading to enhanced CDC. Furthermore, it has been found that the presence of galactose residues in IgG glycans facilitates the formation of IgG hexamers by influencing Fc conformation and enhancing the Fc‐Fc interactions necessary for stable hexamer formation.^100^, ^101^ However, it has been shown that IgG hexamers containing higher levels of galactosylated IgG glycans do not exhibit increased binding to the C1q component of complement compared to hexamers with lower levels of galactosylation. This indicates that the improved binding of hexamers to C1q is due to the higher avidity provided by multiple Fc portions in the IgG hexamer interacting with the C1q component, and that the presence of galactose in IgG glycans, aside from its role in hexamerization, does not affect binding to C1q. On the other hand, it has been observed that monomeric IgG and IgG with a lower propensity for hexamerization exhibit increased binding to C1q when galactose is present in the IgG glycans.^100^, ^101^

Besides the demonstrated role of IgG glycans with galactose in enhancing classical complement activation, one study has shown that galactosylated IgG immune complexes (ICs) can inhibit complement‐mediated inflammation.^102^ Specifically, it has been shown that IgG ICs containing galactosylated IgG glycans are necessary for the association between the FcγRIIb receptor and Dectin‐1 expressed on some immune cells, including neutrophils, which then cooperatively lead to the inhibition of C5a‐mediated pro‐inflammatory immune response. Another study provided further insight, revealing that the anti‐inflammatory activity mediated by galactosylated IgG ICs, FcγRIIb, and Dectin‐1 is also dependent on Galectin‐3—a lectin that recognizes and interacts with galactose residues^103^ (Figure 3). It has been shown that Galectin‐3 binds specifically to galactosylated IgG ICs, but not to non‐galactosylated IgG ICs, and plays a role in cross‐linking these galactosylated IgG ICs with FcγRIIb and Dectin‐1. Therefore, research indicates that IgG glycans with galactose play a role in both pro‐inflammatory and anti‐inflammatory immune responses, and that their involvement in these immune responses depends on the expression and presence of other components involved in the immune response, such as lectins and complement components, as well as on the type of effector cells involved and the type of receptors and their interacting proteins expressed on effector cells.

Numerous studies have shown that in inflammatory and autoimmune diseases, the abundance of IgG glycans lacking galactose increases, while the abundance of IgG glycans with galactose decreases.^104^ Additionally, it has been found that IgG antibodies from arthritic mice are more effective at transferring disease to healthy recipient mice if the IgG antibodies lack galactose in their glycans, compared to unmodified antibodies.^105^ Based on this observation, IgG antibodies with glycans lacking galactose are considered pro‐inflammatory, while IgG antibodies containing galactose in their glycans are characterized as anti‐inflammatory. It was also suggested that galactose‐deficient IgG antibodies elicit a pro‐inflammatory immune response through interaction with mannose‐binding lectin (MBL) and activation of lectin complement pathway.^106^ MBL is the first component of the lectin complement pathway and is known to bind not only to mannose residues but also to exposed GalNAc residues, though it does not bind to galactose residues. IgG antibodies which lack galactose have exposed terminal GalNAc residues, and in vitro studies have shown that these galactose‐deficient IgG antibodies bind to MBL and activate complement.^106^ However, an in vivo study with MBL‐null mice demonstrated that galactose‐deficient IgG antibodies from arthritic animals can induce arthritis in both mice that lack MBL and wild‐type mice.^107^ The findings of this study indicate that the pro‐inflammatory activity of IgG antibodies lacking galactose depends on activating Fcγ receptors and not on MBL.

The observations that IgG glycans lacking galactose are found at higher levels in inflammatory and autoimmune diseases seem to contradict the observed effect of galactose‐containing IgG glycans on enhancing IgG effector function such as ADCC and complement activation, achieved through enhances binding of galactosylated IgG to FcγRIIIa and to the C1q component of the complement system. However, one proposed theory suggests that this contradiction can be reconciled by considering not only the effect of galactose on the potency of pathogenic IgG antibodies, such as autoantibodies, to enhance immune activation but also the effect of galactose on the function of total endogenous circulating non‐specific IgG antibodies.^96^ Based on observations that the galactosylation level of total plasma IgG is elevated in healthy individuals compared to those with autoimmune diseases as well as in individuals with certain autoimmune diseases during remission (such as in remission of rheumatoid arthritis during pregnancy),^104^, ^108^, ^109^ the proposed theory^96^ suggests that: (1) the increased galactosylation level provides non‐specific endogenous IgG with a greater affinity for FcγR and the C1q component of complement; (2) because the quantity of non‐specific endogenous IgG exceeds that of pathogenic IgG autoantibodies, and due to the enhanced binding affinities resulting from increased galactosylation, many FcγR and C1q molecules become occupied by non‐specific IgG, making them less available for binding by pathogenic IgG autoantibodies; and (3) this reduces the ability of pathogenic antibodies to engage immune effector cells and complement, thereby preventing excessive or inappropriate immune responses. In this way, galactosylated non‐specific endogenous IgG helps maintain homeostasis and also supports disease remission. On the other hand, during active inflammatory and autoimmune diseases, the level of glycans lacking galactose on total plasma IgG increases and is also observed that relapses in patients with certain autoimmune diseases are accompanied by a decrease in total IgG galactosylation.^104^, ^110^ Thus, the same theory suggests^96^ that when the level of IgG glycans with galactose is reduced, non‐specific endogenous IgG loses its capacity to provide blocking effects, resulting in decreased effectiveness of the immune system in controlling the disease.

Besides the proposed blocking effects of endogenous non‐specific circulating IgG with galactosylated glycans,^96^ there seem to be additional mechanisms through which endogenous non‐specific IgG might be involved in controlling unwanted immunological responses. For instance, as previously described, galactosylated endogenous IgG could interact with Galectin‐3 to trigger an anti‐inflammatory response mediated by inhibitory FcγRIIIa and Dectin‐1, serving as a mechanism to control the pro‐inflammatory immune response induced by activated complement.^103^

Moreover, a recent study suggests that galactosylated, non‐core‐fucosylated IgG antibodies might be an active component of the anti‐inflammatory effects of intravenous immunoglobulin (IVIg) therapy.^111^ IVIg is an IgG‐enriched preparation derived from pooled serum of thousands of healthy donors. IVIg is used both as a replacement therapy for immunocompromised patients at lower doses (0.4–0.6 g/kg) and as an anti‐inflammatory therapy at higher doses (1–3 g/kg) for various autoimmune and inflammatory conditions, including idiopathic thrombocytopenic purpura (ITP), Guillain–Barré syndrome, Kawasaki disease, and chronic inflammatory demyelinating polyneuropathy (CIDP).^112^, ^113^ The proposed mechanism by which galactosylated non‐core‐fucosylated IgG antibodies in IVIg exert anti‐inflammatory activity is fundamentally similar to the previously described proposed mechanism through which endogenous non‐specific IgG restricts the activity of pathogenic autoantibodies. Specifically, it is proposed that the anti‐inflammatory activity of both galactosylated non‐core‐fucosylated IgG in IVIg and galactosylated endogenous IgG antibodies is achieved by occupying Fcγ receptors, which pathogenic autoantibodies use to activate immune responses such as ADCC, involved in the pathogenesis autoimmune diseases.^96^, ^111^ This recent study demonstrated that a preparation containing galactosylated non‐core‐fucosylated IgG antibodies was more effective at reducing arthritis scores and inflammation in arthritic mice than IgG preparations with other glycosylation profiles, including those with sialylated IgGs.^111^ Additionally, it proved more effective than using 10 times higher doses of standard IVIg.

Overall, it is clear that further research is needed to clarify the role of both galactosylated and non‐galactosylated IgG antibodies in modulating pro‐inflammatory and anti‐inflammatory immune responses.

### 5.4 Effect of IgG glycans with sialic acid on IgG effector functions

Investigations into how the presence of sialic acid in glycans attached to the Fc portion of IgG antibodies affects the interaction of IgG with the C1q component of the complement system, as well as subsequent complement activation, deposition, and CDC efficiency, have yielded inconsistent findings. Two studies have shown that, compared to galactosylated IgG (IgG with glycans in which terminal galactose residues are not modified by sialic acid), sialylated IgG (IgG with glycans that have sialic acids bound to galactoses) exhibit slightly increased binding to the C1q component of complement.^68^, ^100^ One of these studies also showed that the presence of sialic acid in IgG glycans enhanced complement deposition but had little effect on the increase of CDC.^68^ Another study, however, showed that sialylated IgG did not increase complement deposition despite slightly increased C1q binding.^100^ One additional study found that further modification of galactosylated IgG glycans with sialic acid had neither a positive nor a negative effect on the already enhanced binding affinity between IgG and the C1q component achieved through the addition of galactose.^44^ Conversely, there was also a study showing that IgG antibodies containing sialic acid in their glycans had significantly reduced binding to C1q, which resulted in decreased CDC compared to IgG antibodies with glycans containing galactose but lacking sialic acid.^114^

Studies that examined the effect of sialic acid in IgG glycans on the interaction of IgG with different Fcγ receptors (FcγRs) and the activation of downstream effector functions, such as ADCC, have mainly revealed that IgG with sialylated glycans either exhibits the same binding affinity to FcγRs (shown for FcγRIIa/b and FcγRIIIa) as IgG with galactosylated glycans^98^, ^114^ or exhibit only a slight reduction in affinity for FcγRs (shown for FcγRIIIa/b).^2^, ^43^, ^44^, ^68^ These studies also observed that unaffected or slightly decreased binding to FcγRIIIa correlated with ADCC activity that was either unaffected or only slightly decreased in response to sialylated IgG.^2^, ^44^, ^68^, ^98^, ^114^ However, several studies showed different results. Two studies reported several‐fold reductions in both FcγRIIIa and FcγRIIb binding affinities for highly sialylated IgG antibodies compared to IgG antibodies with lower levels of sialylation, along with a corresponding several‐fold reduction in ADCC.^115^, ^116^ Additionally, two other studies found increased binding of sialylated IgG to FcγRs.^67^, ^98^ One study reported this increase in binding specifically for FcγRIIa,^98^ while another study noted a slight increase in binding for FcγRIIa/b and FcγRIIIa/b, but only for IgG antibodies that lacked core fucose in their glycans.^67^

Although contradictory results make it difficult to determine the precise effect of sialylation on IgG‐mediated effector functions activated through FcγRs, it appears that, overall, sialylated IgGs do not significantly differ from galactosylated IgGs in how they influence effector function activation through FcγRs and the efficacy of these functions.

Some other studies, especially those aimed at uncovering the key components and mechanisms responsible for the anti‐inflammatory activity of IVIg therapy, have revealed that the presence of sialic acid in IgG Fc glycans imparts anti‐inflammatory activity to IgG (Figure 3). It has been shown that sialylated Fc fragments (as evidenced by using sialic acid‐enriched Fc fragments derived from IVIg through enzyme digestion and sialylated recombinant Fc fragments) are sufficient to reduce inflammation and alleviate disease symptoms in various autoimmune models.^115^, ^117^, ^118^, ^119^ When sialic acid is removed from the Fc fragments derived from IVIg, their ability to reduce inflammation is significantly reduced.^118^ Moreover, it was found that doses of sialylated Fc fragments that are ten times smaller can lead to a reduction in inflammation comparable to that achieved with the full IVIg preparation.

Investigations into the mechanisms through which sialylated IgG antibodies induce the anti‐inflammatory immune response have led to the identification of several receptors on human cells to which sialylated IgG binds.^118^, ^119^ It was revealed that the anti‐inflammatory effector function of sialylated IgG is activated not by binding to classical FcγRs, but by binding to non‐classical Fc receptors known as Type 2 Fc receptors (Figure 3). To date, the identified receptors for sialylated IgG include the C‐type lectin DC‐SIGN (Dendritic Cell‐Specific Intercellular adhesion molecule‐3‐Grabbing Non‐integrin), which is expressed on regulatory macrophages, dendritic cells, and some monocytes^120^, ^121^, ^122^; the Siglec family member CD22, which is expressed on B cells^123^; and the C‐type lectin DCIR, which is expressed on various immune cells, including dendritic cells, monocytes, and B cells.^124^ In addition, research into the antibody response to the influenza vaccine has identified C‐type lectin CD23, expressed on B cells, but also on various other cells, including monocytes, follicular dendritic cells, platelets, and eosinophils, as a receptor for sialylated IgG.^125^

By binding to these Type 2 Fc receptors, sialylated IgG triggers anti‐inflammatory pathways to suppress inflammation. Specifically, the binding of sialylated IgG to the DC‐SIGN receptor on myeloid‐derived cells induces the production of the IL‐13 cytokine, which subsequently induces the production of the IL‐4 cytokine by basophils.^121^ The IL‐4 cytokine then increases the expression of the inhibitory Fc receptor FcγRIIb on macrophages. The increased levels of FcγRIIb on macrophages raise the threshold for effector macrophage activation by autoantibody immune complexes, thereby reducing inflammation. Of note, some studies have questioned whether DC‐SIGN actually functions as a receptor for sialylated IgG. One study found no interaction between IgG and DC‐SIGN,^126^ while another study reported no difference in binding affinity for DC‐SIGN between differentially glycosylated IgGs and that interaction with DC‐SIGN is mediated by Fab portion of IgG rather than the Fc portion.^127^ Furthermore, it has been demonstrated that sialylated IgG binding to the CD22 receptor on B cells modulates the cell fate signals conveyed through the B‐cell receptor (BCR) signaling pathway, leading to the apoptosis of B cells.^123^ This may also represent a mechanism by which sialylated IgG suppresses autoimmunity, potentially by reducing the survival of pathogenic B cells at one of the stages, including pre‐germinal center (GC) B cells, GC B cells, or plasma cells.^128^ Another way sialylated IgGs may exert anti‐inflammatory effects involves their binding to the DCIR receptor which leads to activation of regulatory T cells that suppress inflammation.^124^

That sialylation can act as a key switch between pro‐inflammatory and anti‐inflammatory IgG effector functions is well demonstrated by research on pathogenic IgG autoantibodies. These studies show that sialylation not only reduces the pathogenicity of these antibodies but also that sialylated pathogenic antibodies can lead to improvement of active disease.^129^ This effect has been observed not only when pathogenic antibodies are sialylated in vitro and introduced into autoimmune disease model mice,^130^ but also when pathogenic antibodies are specifically sialylated in vivo, converting them into anti‐inflammatory mediators that reduce autoimmune inflammation.^131^ These findings hold significant therapeutic potential.

In the context of the significance of sialylated IgG for IVIg therapy, it is important to understand that IVIg exerts anti‐inflammatory effects through various mechanisms, with only a subset of these mechanisms appearing to rely on IgG sialylation.^132^, ^133^, ^134^

### 5.5 Effect of high‐mannose IgG glycans on IgG effector functions

Although high‐mannose IgG glycans are not very relevant for human plasma IgG because of their low levels (<1% of total plasma IgG glycans),^11^, ^13^ they can be significant for the properties of recombinant antibodies, where it is observed that their levels can sometimes exceed 20%.^135^

It has been observed that high‐mannose IgG glycans have a higher affinity for the FcγRIIIa receptor compared to core‐fucosylated forms of complex and hybrid glycans.^44^, ^65^ Additionally, their binding affinity for FcγRIIIa and ADCC activity is comparable to, or slightly lower than, that of non‐core‐fucosylated complex and hybrid glycans (Figure 3). This observation that IgG with high‐mannose glycans has a high affinity for FcγRIIIa and induces high ADCC activity is probably related to the fact that high‐mannose glycans attached to IgG do not contain core fucose. Therefore, the absence of core fucose, rather than the presence of mannose residues, may be responsible for the higher FcγRIIIa binding and enhanced ADCC observed for IgG containing high‐mannose glycans.^136^ However, IgG with high‐mannose glycans suffers from problems such as more rapid clearance^135^, ^137^ and increased immunogenicity^138^ compared to IgG with complex type of glycans. These issues limit their use as a strategy to improve ADCC, for example in monoclonal antibody therapy.

Regarding the effect of high‐mannose glycans on IgG binding to the C1q component of complement and the activation of CDC, it has been observed that IgG with both hybrid and high‐mannose N‐glycans show lower binding to C1q and induce lower CDC activity compared to IgG with complex type N‐glycans.^44^, ^65^

## 6 ROLE OF OTHER IgG GLYCANS OUTSIDE THE CONSERVED ASN 297 GLYCOSYLATION SITE

### 6.1 Role of glycans found in the IgG Fab variable domain

As previously mentioned, 15%–25% of IgG antibodies in the plasma of healthy individuals contain glycans attached to their Fab variable domain.^139^ Research on Fab glycans has identified several important roles of these glycans, and further research is anticipated to yield new insights.

Fab glycans have been shown to influence antigen binding.^139^, ^140^, ^141^, ^142^ They can enhance, decrease, or leave antigen binding unaffected, depending on the specific antibody. It appears that Fab glycans attached to naturally occurring glycosylation sites in antibodies that have undergone in vivo affinity selection mainly have a positive influence on antigen binding.^141^ In contrast, the introduction of glycosylation sites in vitro generally negatively affects antigen binding. In studies on the effect of Fab glycans present on anticitrullinated protein (auto)antibodies (ACPAs), it has shown that presence of Fab glycans is associated with overall reduced binding affinity for autoantigens.^142^ Removal of sialic acid from the Fab glycans in ACPAs enhanced antigen binding. However, a significantly higher binding affinity was achieved by removing the entire Fab glycan, while in the case of one reported ACPA antibody, the removal of Fab glycans decreased antigen binding.^140^, ^142^ It is assumed that this masking of self‐reactivity by Fab glycans plays an important role in processes during which self‐non‐self discrimination is acquired and in the development of self‐reactive immune responses that underlie autoimmunity.^142^, ^143^

It has also been shown that Fab glycans can modulate the specificity of antibodies for antigens.^141^ Specifically, depending on the position of the glycan within the Fab portion of IgG, it is possible to enhance the specificity of an antibody for a particular antigen, thus avoiding cross‐reactivity.

Additionally, naturally occurring Fab glycans seem to enhance the stability of IgG antibodies, and similar effects can be achieved by introducing glycans in vitro to improve the stability of IgG antibodies for research, diagnostic, or therapeutic purposes.^144^

Furthermore, research has shown that Fab glycans affect the interaction between IgG and the neonatal Fc receptor (FcRn), which appears to directly influence IgG's ability to cross the placenta and may also affect its half‐life.^145^ FcRn, located in endothelial cells, regulates the half‐life of IgG by binding IgG and recycling it back into the circulation.^10^, ^145^ This recycling process is responsible for the prolonged half‐life of IgG, which is approximately 3 weeks. This receptor is also responsible for transferring IgG antibodies across the placenta.^10^, ^145^ It has been demonstrated that the presence of Fab glycans in IgG antibodies results in decreased binding to the FcRn receptor expressed on cells compared to IgG antibodies without Fab glycans, likely due to steric hindrance.^145^ Furthermore, the presence of sialic acid in Fab glycans, despite its negative charge, does not have an additional inhibitory effect on this interaction. The study also showed that, due to decreased binding to FcRn, IgG antibodies with Fab glycans are transported across the placenta 20% less efficiently compared to those without Fab glycans. Another study found no significant difference in the clearance rates of IgG antibodies with different Fab glycan compositions, except for a slightly faster clearance of those IgGs with Fab glycans that lack galactose.^146^ Of note, although the FcRn interaction site is located within the Fc portion of IgG, it is specifically positioned at the CH2‐CH3 domain interface, and Fc glycans do not seem to influence the interaction between FcRn and IgG.^147^ Evidence shows that IgG without Fc glycans has similar clearance and half‐life as normally glycosylated IgG.^148^ Furthermore, while some in vitro studies suggest that IgGs containing Fc glycans with two galactoses and glycans with sialic acid have enhanced binding to FcRn,^43^, ^44^ these differences do not appear to be significant under physiological conditions when the effects on IgG placental transport or in vivo half‐life are considered. Specifically, no difference in Fc glycosylation between maternal and fetal IgG,^149^ nor in the in vivo half‐life of IgGs with different Fc glycosylation patterns,^150^ has been observed.

Evidence also suggests that Fab glycans play a role in the some of the observed anti‐inflammatory effects of IVIg therapy.^139^, ^151^

### 6.2 Role of hinge O‐glycans and CH3 N‐glycans in the IgG3 subclass

The role of O‐glycans attached to the hinge region of the IgG3 subclass, as well as the role of N‐glycans attached to the Asn 392 glycosylation site, is almost completely unexplored. Therefore, the roles described here are mostly speculative.

Based on the observation that IgG3 hinge peptides with attached O‐glycans (O‐glycopeptides) were resistant to cleavage by endoprotease AspN, in contrast to IgG3 hinge peptides of the same sequence that lacked glycans,^31^ it is hypothesized that O‐glycans protect the hinge region of IgG3 from protease cleavage.

It is also theorized that O‐glycans support the hinge region in adopting an extended conformation,^31^ which could account for the larger Fab–Fab distance and greater flexibility observed in IgG3 compared to other IgG subclasses.^152^

Furthermore, one study observed that in IgG immune complexes (IgG‐ICs) from patients with rheumatoid arthritis, the levels of exposed terminal galactose and GalNAc residues in O‐glycans were higher compared to controls but decreased after therapy, although they remained above the levels found in controls.^153^ Given this finding, along with evidence that galactose‐deficient O‐glycans on IgA are implicated in IgA nephropathy,^154^ it is supposed that changes in O‐glycans on IgG3 may play a role in the development or progression of certain diseases.^31^

The potential role of N‐glycans at the Asn 392 glycosylation site in the CH3 domain of some IgG3 allotypes is even less understood than the role of IgG3 O‐glycans. It has been suggested that these N‐glycans could influence the interaction between the two CH3 domains and affect the dissociation of the CH3 domains.^30^, ^155^

### 6.3 Effect of glycosylation on IgG B‐cell receptor (IgG‐BCR) function

The B‐cell receptor (BCR) is a transmembrane protein expressed on the surface of B cells.^156^ Signaling through this receptor is crucial for B‐cell development and differentiation. Another very important role of this receptor is that it binds antigens, which then triggers B‐cell activation. Activated B cells expressing BCR then enter germinal centers (GCs) where they proliferate, undergo affinity maturation, and differentiate into plasma cells, that produce high‐affinity antibodies, and memory B cells. B cells found in GCs often express the IgG isotype of the B‐cell receptor (BCR), though they can also express BCRs of other immunoglobulin isotypes. The structure of the IgG‐BCR is very similar to that of secreted IgG, with some additions.^156^ It consists of the membrane‐bound IgG protein, which has additional transmembrane helices, and the Igα/Igβ heterodimer to which the IgG protein is non‐covalently bound. The role of the IgG protein part is to bind antigens, while the heterodimer transduces the signal upon antigen binding. Given the structural similarity between IgG‐BCR and secreted IgG, and the known importance of Fc‐linked glycans and glycans in the Fab portion of IgG in determining the function and properties of secreted IgG, studies have also investigated the roles of glycans in the Fc and Fab portions of IgG‐BCR, to determine whether IgG glycans also play a role in the events shortly after antigen recognition by B cells and during the affinity maturation of GC‐B cells, rather than only influencing function of secreted IgG produced by terminally differentiated plasma cells.^142^, ^157^

While Fc glycans in secreted IgG antibodies are well‐recognized for their role in enabling and modulating various effector functions of IgG, research indicates that glycans present in the Fc portion of IgG‐BCRs expressed on GC‐derived B‐cell line do not affect IgG‐BCR function.^157^ Specifically, no effects of Fc glycans on the surface expression of IgG‐BCRs, antigen binding, BCR downregulation after antigen binding, or BCR signal transduction were observed. On the other hand, it has been shown that glycans in the Fab portion of IgG‐BCRs have a significant impact on IgG‐BCR function.^142^ Fab glycans on IgG‐BCR have been shown to modulate antigen binding, enhance B‐cell activation, and reduce BCR downregulation, thereby preserving higher levels of IgG‐BCR expressed on the cell surface following antigen binding.

## 7 UNDERSTANDING IgG GLYCOSYLATION THROUGH STUDIES IN THE GENERAL POPULATION

The emergence of high‐throughput methods for IgG glycosylation analysis, starting in the late 2000s, has made it possible to investigate IgG glycosylation in large sample sizes involving several thousand individuals from both the general population and specific disease cohorts.^12^, ^21^ This advancement has enabled a comprehensive and detailed exploration of IgG glycosylation characteristics in both general populations and specific diseases, providing new insights with greater statistical validity.

### 7.1 Variability of IgG glycosylation and the genetic and environmental influences on this variability

Plasma IgG glycosylation has been analyzed in several large‐scale studies. Early studies focused on individuals from various European populations, while a later study included individuals from different global populations, all representing the general population (not selected based on specific diseases). A 2011 study examined around 2000 individuals from three different European populations,^13^ a 2014 study included over 5000 individuals from four European populations,^158^ and a 2020 study analyzed over 2500 individuals from 27 populations worldwide.^20^ These studies revealed that there is high interindividual variability in IgG glycosylation. It has been shown that the level of glycans without galactose found on plasma IgG antibodies among different individuals can vary from around 15% to 65%, the level of IgG glycans with one galactose can vary from around 25% to 45%, the level of IgG glycans with two galactoses, as well as the level of IgG glycans that contain sialic acid, can range from around 5% to 30%, and the level of IgG glycans with bisecting GlcNAc can vary from around 7% to 25%.^13^, ^20^

The heritability analysis from the twin study indicates that genetic factors explain approximately 50% of the differences in IgG glycosylation observed between individuals.^159^ The other half of the variation is attributed to a range of physiological and environmental factors, including hormonal differences and lifestyle behaviors (like diet and physical activity), which shape the glycosylation of IgG in each person. For instance, research has indicated that the levels of glycans without galactose, with one galactose, and with two galactoses are associated with developmental indices which are standardized statistical measures that assess and compare quality of life across various countries.^20^ It has also been demonstrated that the levels of some IgG glycans are influenced more strongly by genetic factors than others. Specifically, the levels of IgG glycans that lack galactose or contain only one galactose are highly heritable, with a heritability estimate of around 70%. In contrast, the heritability of other IgG glycans—those with two galactoses, those containing sialic acid, and those with bisecting GlcNAc—is estimated to be about 40% or lower.^159^

Of note, a study analyzing IgG glycosylation in 95 different Collaborative Cross (CC) mouse strains—created from eight founder strains (five classical inbred and three wild‐derived) through three generations of interbreeding, followed by at least 20 generations of inbreeding—found that this breeding strategy produces variability in IgG glycosylation comparable to that seen in human populations.^160^ The study also identified several strains with higher levels of IgG glycans containing bisecting GlcNAc. The role of these glycans is not well understood, but they have been shown to be changed in many diseases.^104^ This finding could facilitate research into their role, as bisecting GlcNAc glycans are usually very low or undetectable in classical laboratory mouse strains, which has previously limited research on these glycans in such models.

### 7.2 Characteristics of IgG glycosylation in relation to age and sex

Already very early studies on IgG glycosylation in humans observed that IgG glycosylation changes significantly with age.^161^ The most notable changes with age are seen in the levels of glycans lacking galactose, which increase with age, and glycans with two galactoses, which decrease with age.^20^, ^158^ Additionally, the levels of glycans with sialic acid and bisecting GlcNAc also change with age, decreasing and increasing, respectively. These changes in IgG glycosylation with age are observed in both women and men.^20^, ^158^ Thus, all the aforementioned assessments of genetic and environmental contributions to IgG variability have been adjusted for the effects of age.^159^ Studies have demonstrated that age can account for approximately 20%–35% of the variability in the levels of IgG glycans lacking galactose and around 20%–30% of the variability in the levels of IgG glycans with two galactoses, depending on the studied populations.^13^, ^20^ In contrast, sex is not a major predictor of IgG glycan levels, explaining only about 1% of the variability in glycans lacking galactose and those with two galactoses.^13^, ^20^ Nevertheless, there are some observed differences in IgG glycosylation between sexes that should be noted. Women generally have higher levels of glycans with two galactoses and lower levels of glycans without galactose than men before age 55.^14^, ^20^ After age 55, however, the levels of these glycans become similar between men and women. Furthermore, while the levels of glycans lacking galactose, glycans with two galactoses, and glycans with one sialic acid change at a relatively consistent rate throughout adulthood in men, either increasing or decreasing linearly, depending on IgG glycan, women experience more pronounced changes in these IgG glycan levels between ages 45 and 55, a period of life that coincides with the menopausal transition or perimenopause. More specifically, it has been demonstrated that, before perimenopause, the levels of IgG glycans lacking galactose, glycans with two galactoses, and glycans with one sialic acid remain relatively stable in women.^162^ However, during perimenopause, there is a notable increase in the levels of glycans without galactose and a decrease in the levels of glycans with two galactoses and those with one sialic acid. Once women enter menopause, these glycans continue to change in the same direction as during perimenopause, but at a much slower rate.^162^ These findings, along with other research, indicate that IgG glycan levels are significantly affected by hormonal changes, especially by estrogen.^162^, ^163^ The results of the study that investigated changes in IgG glycosylation in nearly 2000 women in different stages—premenopausal, perimenopausal, and menopausal women—also indicate the potential of IgG glycans as a biomarker for perimenopause.^162^

Notably, changes in IgG glycosylation have been observed during pregnancy, likely driven by hormonal changes. This was demonstrated in a study comparing glycosylation during pregnancy to post‐delivery in 29 women, with separate analyses of the Fab and Fc portions of IgG.^11^ The study revealed differences in glycosylation changes associated with pregnancy between the Fab and Fc portions of IgG. On one hand, it is understandable that these differences may occur due to the distinct roles of the Fab and Fc portions of IgG—Fab is responsible for antigen binding, while Fc has effector functions. On the other hand, it is intriguing to explore how these glycosylation differences arise between the two portions of the same protein. This could be related to the accessibility of each portion to glycosylation enzymes, or it might involve more complex regulatory mechanisms.

Turning back to the changes in IgG glycosylation with age—increased levels of glycans lacking galactose and decreased levels of those with galactose and sialic acid—it is important to understand how these changes might affect IgG's role in immune responses as an individual ages. As previously explained, IgG's ability to mediate pro‐inflammatory or anti‐inflammatory responses depends largely on its glycosylation.^46^ Since similar glycosylation changes that occur with aging are also seen in various inflammatory diseases, this suggests that these age‐related glycosylation changes likely shift IgG's role toward promoting inflammation.^104^ Furthermore, these age‐related glycosylation changes are thought to make IgG act as a pro‐inflammatory mediator, contributing to the development of the state of low‐grade inflammation with age, known as “inflammaging,” which is considered a driver of aging and is associated with the development of age‐related diseases.^164^, ^165^ Thus, IgG glycans are viewed as one of the molecular effectors of the aging process.

Given that IgG glycosylation changes with age and that IgG glycans are considered an important factor in the aging process,^164^, ^165^ and since it has been shown that IgG glycan levels are influenced by both genetic and environmental factors,^159^ the potential of IgG glycans as biomarkers of aging to help distinguish between healthy and unhealthy aging patterns has been investigated.^158^ Studies have shown that IgG glycans have a strong correlation with chronological age and can account for 41% to 58% of the variation in age, depending on the population studied and the specific IgG glycans included in the models.^158^, ^166^ For example, it has been demonstrated that the age calculated using the GlycanAge index, which is based on three specific glycans can explain 58% of the variance in chronological age. Moreover, it has also been demonstrated that the GlycanAge index correlates with physiological and biochemical parameters associated with an unhealthy lifestyle, such as BMI, triglycerides, glucose levels, and waist circumference, after adjusting for age and sex.^158^ These parameters are also linked to an increased risk of age‐related diseases. Therefore, age measured by glycans not only correlates with chronological age but also reflects an individual's overall health status, effectively measuring what is known as biological age. Later research conducted in a cohort of individuals with Down syndrome also demonstrated that IgG glycans can detect accelerated aging, further highlighting their potential as biomarkers of biological age.^167^ These extensive research on changes of IgG glycosylation during aging has paved the way for IgG glycans to be marketed as biomarkers of aging.^13^, ^158^ Biomarkers based on IgG glycans are now recognized as the only commercially available biomarker of biological age, demonstrating their significant value in the field of biomarkers of aging.^168^

## 8 IgG GLYCOSYLATION IN DISEASES

Changes in IgG glycosylation have been observed in a wide range of diseases, including inflammatory and autoimmune conditions, infectious diseases, cardiometabolic disorders, cancers, neurodegenerative diseases, and many other diseases.^104^

### 8.1 IgG glycosylation in inflammatory and autoimmune diseases

Research on IgG glycosylation in healthy individuals has shown that IgG glycosylation correlates with levels of C‐reactive protein (CRP) and interleukin‐6 (IL‐6), two common inflammation markers.^22^ Specifically, lower levels of galactosylation and sialylation, as well as higher levels of core fucosylation, have been associated with increased CRP and IL‐6 levels. Alterations in IgG glycosylation are seen in many inflammatory and autoimmune diseases, including prevalent autoimmune diseases such as rheumatoid arthritis (RA), systemic lupus erythematosus (SLE), inflammatory bowel disease (IBD), and multiple sclerosis (MS).^104^ In nearly all inflammatory and autoimmune diseases, a similar IgG glycosylation pattern is observed, characterized by decreased levels of glycans with galactose and sialic acid and increased levels of IgG glycans without galactose.

Among autoimmune diseases, IgG glycosylation changes have been most extensively studied in rheumatoid arthritis (RA). Lower levels of galactosylated IgG glycans and/or increased levels of agalactosylated IgG glycans, compared to controls, have been consistently found in RA patients. However, some studies have also reported decreased levels of sialylated IgG glycans,^169^, ^170^ and increased levels of IgG glycans with bisecting GlcNAc^171^ and fucosylated IgG glycans^170^ compared to healthy controls. Interestingly, several studies have found changes in total plasma IgG glycosylation, specifically increased levels of agalactosylated glycans, years before the onset of RA symptoms. One study observed these changes up to 10 years (median 4.3 years) before symptoms appeared,^172^ while another study identified them at least 3.5 years before RA diagnosis^173^ when compared to IgG glycan levels in healthy individuals. Additionally, patients with early synovitis who later developed RA had different IgG glycosylation 2 years before RA diagnosis compared to those who developed other types of inflammatory joint diseases.^174^ These studies suggest that IgG glycosylation changes occur early in disease development as an early molecular marker of RA and might even play a role in driving the disease. One study, however, found that in ANCA‐positive patients with joint pain, those who later developed RA exhibited changes in the levels of galactosylated IgG glycans, but these changes were observed exclusively on ACPA‐specific IgG, occurring 3 months before the onset of the disease.^175^ These findings highlight the value of monitoring changes in both the glycosylation of total IgG and (auto)antigen‐specific IgG as early indicators of the disease. Studies also found that in RA, changes in the levels of galactosylated IgG glycans not only precede disease onset but are also associated with disease activity and outcomes in diagnosed patients. Higher levels of galactosylated glycans and lower levels of agalactosylated glycans correlated with less severe disease activity^171^, ^173^ and a more favorable prognosis observed within a 2‐year period following RA diagnosis.^176^ It should be mentioned that the correlation between RA disease activity and the levels of galactosylated IgG glycans has been demonstrated specifically for Fc glycans.^171^ Regarding Fab glycans, it has been shown that patients with RA exhibit higher levels of Fab glycosylation compared to controls, but no correlation between Fab glycosylation and disease activity has been observed. Additionally, research have shown that levels of galactosylated IgG glycans increase in RA patients in response to methotrexate (MTX) treatment, whether used alone or combined with Remicade (a chimeric anti‐TNF‐α antibody).^170^, ^177^ Moreover, it has been shown that the ratio of agalactosylated to galactosylated IgG glycans can help predict whether a patient will respond to MTX therapy,^178^ making IgG glycans a potentially valuable tool in RA treatment management.

Regarding changes in IgG glycosylation in systemic lupus erythematosus (SLE), a study that investigated IgG glycosylation in patients from three distinct populations—African Caribbeans, Latin Americans, and Han Chinese—revealed alterations in the levels of various complex IgG glycans.^179^ In SLE patients, there was an increase in glycans lacking galactose and those with bisecting GlcNAc, and a decrease in glycans containing galactose, sialic acid, and core fucose. This study also demonstrated that these glycan changes could help differentiate between patients with more severe forms of the disease and those with less severe forms.

Research on IBD patients has shown that both Crohn's disease (CD) and ulcerative colitis (UC) patients exhibit increased levels of agalactosylated IgG glycans and reduced levels of mono‐ and digalactosylated, as well as sialylated IgG glycans.^180^, ^181^ However, these changes were more pronounced in CD patients, suggesting that IgG glycosylation plays a more significant role in the mechanism of CD compared to UC. A very recent study^182^ supports this, showing that an increase in the level of agalactosylated IgG glycans is observed in the plasma of individuals who will develop Crohn's disease (CD) up to 6 years before the onset of the disease, while in future UC patients, such changes that precede disease onset are not observed. The same study also revealed that the agalactosylated form of antibodies directed against mannose are pathogenic and contribute to the early stages of intestinal inflammation during the preclinical phase of the disease. Given that IgG glycosylation changes are more pronounced in CD compared to UC, and that differences in the levels of fucosylated glycans and glycans with bisecting GlcNAc have been observed between CD and UC patients, it has been demonstrated that IgG glycans can be used to differentiate between these two conditions.^180^, ^181^ This indicates potential of IgG glycans as diagnostic tools in IBD.

In MS, IgG glycosylation changes have shown to be more pronounced in cerebrospinal fluid than in plasma,^183^, ^184^ which is expected given the pathogenesis and localization of the disease process in MS.

In addition, based on observations in autoimmune disease PR3‐ANCA associated vasculitis it appears that monitoring IgG glycan levels can also aid in predicting relapses.^110^, ^185^

### 8.2 IgG glycosylation in cardiovascular diseases

Inflammation is an important component of many cardiovascular diseases (CVD) and, considering the importance for IgG glycans for the regulation of inflammation, it is not surprising that changes in IgG glycosylation were observed in many studies. The initial study reporting the link between IgG glycosylation and cardiovascular diseases was a cross‐sectional comparison of the cardiovascular disease risk score and IgG glycome composition in two large UK cohorts.^186^ The observed associations were mainly with galactosylation (which associated with lower risk) and bisecting GlcNAc, which positively correlated with the cardiovascular disease risk score. These associations essentially replicated in a Chinese population in a subsequent study.^187^ The analysis of the total plasma glycome in the 2500 individuals from the German subset of the EPIC cohort revealed that one of these glycans (biantennary glycan with core fucose and galactose on the 3‐arm, labeled GP5 in that study) was the best predictor of future CVD events in women.^188^ Subsequently, the IgG glycome was analyzed in the same cohort and the association of the same glycan structure (labeled GP9 in that study) with future CVD events in women was again observed, confirming that this glycan in the plasma glycome indeed originated from IgG.^189^ Interestingly, although the strength of association with incident CVD was comparable in men, the predictive power was contained in different IgG glycans, suggesting that IgG glycome has some sex‐specific roles in the development of CVD. These associations were recently replicated in two more prospective cohorts, with comparable hazard ratios.^190^ Due to small number of incident cases, sex‐stratification was not performed in that study, but the fact that much weaker association with GP9 was observed in the TNT cohort, which had only 16% of women, is supportive of previously observed sex‐specific effects. Although causality cannot be determined from these studies, one mice study suggested that dietary supplementation with *N*‐acetylmannosamine (ManNAc, precursor of sialic acid), which resulted in the increased IgG sialylation, protected obese mice from developing hypertension.^191^ Differences in IgG glycome composition strongly correlated with the expected lifespan in different human populations,^20^ but mechanistic aspects that underly these associations are not known.

### 8.3 IgG glycosylation in infectious diseases

Glycosylation of IgG antibodies has been investigated in infectious diseases^104^ and in response to vaccination,^192^ though not as extensively as in inflammatory and autoimmune conditions. Given the recent COVID‐19 pandemic caused by the SARS‐CoV‐2 virus, it is not surprising that IgG glycosylation has also been thoroughly studied in relation to this disease, both during and after the pandemic. Research on COVID‐19 patients has revealed that the glycosylation patterns of total plasma IgG undergo significant alterations in severe cases. Specifically, in severe COVID‐19 cases, there was an increase in agalactosylated and core‐fucosylated IgG glycans, along with a decrease in galactosylated and sialylated glycans, as well as IgG glycans with bisecting GlcNAc.^193^, ^194^ In contrast, in mild cases, changes in IgG glycosylation were minimal or undetectable and were mostly restricted to changes in the levels of IgG glycans with bisecting GlcNAc.^194^ It has also been demonstrated that the glycosylation profile of total plasma IgG at diagnosis is strongly correlated with disease severity assessed 14 days later.^195^ Higher levels of agalactosylated glycans and glycans with bisecting GlcNAc, along with lower levels of galactosylated and sialylated glycans, have been associated to a worse prognosis. There was also a study that compared changes in total plasma IgG glycosylation during the course of influenza and COVID‐19.^196^ While the levels of most IgG glycans changed similarly in both diseases, changes in (a)galactosylated glycans were observed specifically in deceased COVID‐19 patients. In contrast, patients with moderate and severe COVID‐19 who survived did not show changes in (a)galactosylated glycans, nor did patients with influenza. These studies indicate the potential of measuring total plasma IgG glycosylation at the time of diagnosis and monitoring it throughout the disease course to determine disease severity. They also suggest that IgG glycosylation could be one of the mechanistic factors contributing to disease severity. Further insight into the role of IgG glycosylation in the mechanisms underlying severity in infectious diseases has been gained by studying the glycosylation of antigen‐specific IgG. It has been demonstrated that the response to enveloped viruses, such as SARS‐CoV‐2, is characterized by the production of higher levels of afucosylated antigen‐specific IgG antibodies^197^ and that these afucosylated antibodies can lead to an overactive immune response that is pathological for the patient, due to increased binding to the FcγRIIIa receptor, which activates ADCC mediated by NK cells and other immune effector cells. This mechanism, which contributes to disease severity, appears to be present not only in COVID‐19 patients but also in individuals infected with the dengue virus.^198^ Conversely, in some cases, such as HIV patients who effectively control their disease^199^ and malaria patients,^200^ afucosylated antibodies are observed to aid in better infection control by enhancing the immune response to the pathogen, without causing excessive pathology. These studies indicate that IgG glycosylation can significantly impact disease outcomes, with its effects varying depending on the virus or other pathogens involved. Therefore, understanding the specific glycosylation changes associated with different pathogens is crucial for developing targeted therapeutic strategies and improving patient outcomes.

**FIGURE 4:**
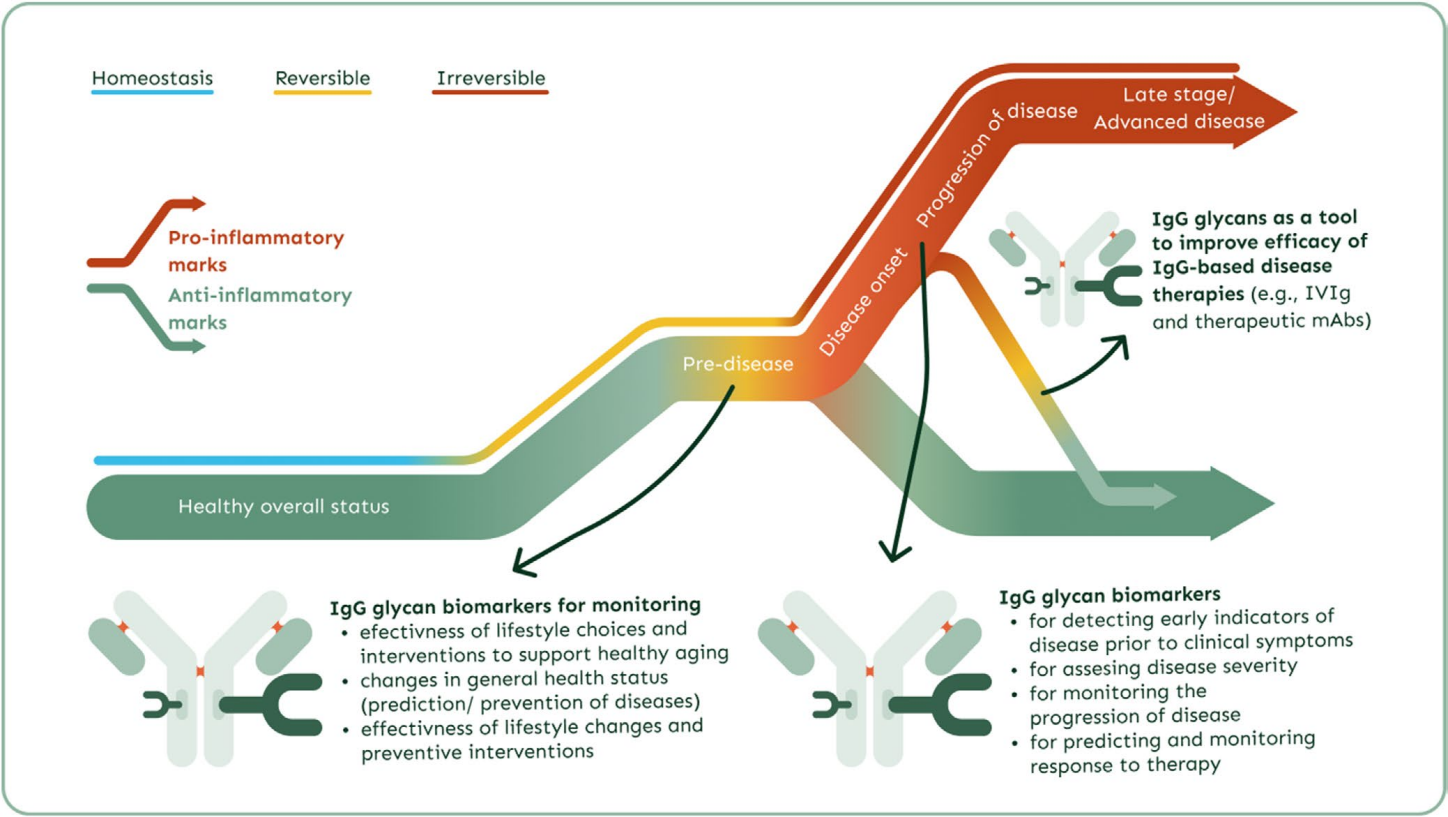
IgG glycans are important biomarkers and functional effectors in health and disease. Changes in IgG glycosylation serve as a measure of alterations in inflammatory status, making IgG glycans useful for monitoring aging trajectories and as indicators of disease status at different stages, including subclinical and early disease, as well as during disease progression. IgG glycans are also recognized as valuable tools for improving the efficacy of IgG‐based therapies, such as therapeutic monoclonal antibodies and IVIg therapy, used in various diseases.

Taken together, extensive research into IgG glycosylation in both healthy populations and various disease cohorts demonstrates the considerable potential and importance of IgG glycans (Figure 4). Throughout the aging process, IgG glycans can be used to monitor and detect changes in overall health status. For example, alterations in glycan patterns may reflect increases in markers associated with inflammation or metabolic disruptions, signaling potential health risks. This could enable early differentiation between healthy and unhealthy aging, allowing for timely interventions to prevent negative aging trajectories and reduce disease risk.

In individuals with existing diseases, IgG glycans can be valuable at different stages of illness (Figure 4). They may serve as early molecular indicators of subclinical disease, potentially identifying issues before clinical symptoms emerge. Additionally, they can contribute to accurate and timely diagnosis, assess disease severity and prognosis in its early phases, and assist in selecting appropriate treatments and monitoring therapeutic responses, ultimately improving overall patient management and outcomes.

## 9 LONGITUDINAL AND INTERVENTION STUDIES OF THE IgG GLYCOME

Large cross‐sectional studies revealed associations between IgG glycome composition and numerous diseases and traits, but large interindividual differences in IgG glycome composition represent a significant limitation for the use of IgG glycans as a potential diagnostic tool. Furthermore, complex regulation of IgG glycosylation and the involvement of numerous genetic, epigenetic, and environmental factors makes any conclusion about causality of the observed associations a challenge. Therefore, longitudinal (with repeated sampling of the same individual) and intervention (with introduction of a specific change between time points) studies are needed to enable deeper understanding of changes in IgG glycome composition that associate with different biological and pathobiological processes.

### 9.1 Effect of body weight and nutrition on IgG glycosylation

The largest longitudinal study of the IgG glycome was performed on the TwinsUK cohort and included 2146 participants of the TwinsUK study, with samples collected at multiple timepoints over a 20‐year period. The comparison of changes in body weight with changes in the IgG glycome composition revealed that losing weight resulted in a “younger,” less pro‐inflammatory IgG glycome composition, while gaining weight resulted in the acceleration of glycan aging in all studies age groups.^201^ Glycan that was the most responsive to weight loss was digalactosylated (G2) that increased 0.2% per 1 kg/m^2^ decrease in BMI (with adjusted p value of 5.85 × 10–06). Agalactosylated (G0) glycans also changed, but with smaller effect (0.1% decrease per 1 kg/m^2^ decrease in BMI) and the adjusted p value was smaller (1.79 × 10–02), suggesting weaker effect. This is consistent with previous studies that estimated much higher heritability of G0 (72%) then G2 glycans (41%).^159^ Single enzyme, ß1,4‐galactosylransferase converts G0 into G2 by two subsequent additions of a galactose residue, which infers that genetic (and other) factors affecting these two glycan classes should have similar effects, but this does not appear to be the case. This is further confirmed by a recent large genome wide association study (GWAS) that identified different genetic loci affecting agalactosylation (G0), monogalactosylation (G1), and digalactosylation (G2) of IgG,^202^ suggesting that regulation of IgG glycosylation is highly elaborated.

Effects of caloric restrictions on the IgG glycome were so far tested in only two studies. In small study of 37 patients preparing for a bariatric surgery, caloric intake was restricted to 900 kcal/day until 20% loss in body mass was achieved (6.54 ± 3.4 months). The only statistically significant change that was observed in that study was a decrease in the bisecting GlcNAc.^201^ Subsequent bariatric surgery resulted in extensive alterations of the IgG glycome that accompanied progressive weight loss during 1‐year follow‐up. Interestingly, after the bariatric surgery the change in G0 was stronger than in G2, and IgG sialylation and monogalactosylation also improved, suggesting that effects of bariatric surgery extend beyond the effects of simple lifestyle‐induced weight loss.^201^ However, the effect of caloric restriction on bisecting GlcNAc did not replicate in a much larger study in which nearly 1000 individuals were restricted to 800 kcal/day for 2 months. In that study, the main effect was a decrease in G0,^203^ the same effect that was observed in twins who were losing weight between the two time points. Following 2 months on low‐calorie diet, participants who achieved to lose 10% of body mass were transferred to one of five different more sustainable “maintenance” diets. Interestingly, on each of the diets nearly the same number of individuals changed levels of individual IgG glycans up and down, resulting in the absence of a significant change at the level of a population. However, the extent of changes was much larger than what is observed without an intervention,^204^ suggesting that each of the different maintenance diets had positive effect on some participants, while negative on others.

### 9.2 Effect of physical activity on IgG glycosylation

Physical activity is essential for the preservation of health and numerous studies clearly demonstrated positive effects of exercise. However, excessive wear and tear is also harmful, which is most obvious in professional athletes. Recently published study that compared IgG glycome compositions in inactive, active, and professional athlete populations showed both positive and negative effects of physical activity. While physically inactive people had higher glycan age than people who were active, the effect in professional athletes was the opposite, resulting in them being comparable to physically inactive, obese individuals.^205^ Another study analyzed IgG glycome in 397 previously inactive individuals that underwent one of three different exercise programs for 3 months.^206^ Unexpectedly, the observed changes in IgG glycosylation reflect an increased pro‐inflammatory IgG potential, suggesting that 3 months are not sufficient for positive effects of physical activity to manifest in the IgG glycome composition. However, in one smaller study on 29 younger individuals, positive effects were observed in a similar time‐frame, suggesting that the effects of different exercise programs can be very individual.^207^

### 9.3 Effect of hormones and drugs on IgG glycosylation

Several pharmacological interventions were reported to have strong effects on IgG glycosylation. So far oestrogens have been identified as the strongest impact.^208^, ^209^ After chemical suppression of gonadal hormones IgG glycome changed considerably in the group receiving placebo hormonal supplementation, while supplementation with oestradiol (in women) or testosterone (in men) prevented this change. However, when aromatase converting testosterone to estrogen was inhibited, the testosterone did not have an effect, indicating that testosterone acts through estrogen even in men.^208^ The same effect was reported in women taking anastrozole (aromatase inhibitor) to treat breast cancer, though the effect was not as strong and was not observed in all participants.^210^ Subsequent more detailed analysis of protein glycosylation revealed extensive changes in IgG galactosylation and sialylation, as well as in the levels of bisecting GlcNAc on IgG. The effects of estrogen depletion are apparently restricted to IgG, since glycosylation of other plasma proteins was not altered in the same way.^163^, ^209^ Longitudinal monitoring of a large number of females during perimenopausal transition revealed the same type of changes, with IgG glycome displaying accelerated aging during the perimenopause period.^162^ Molecular mechanism linking estrogen to IgG glycosylation is still not known, but an initial attempt to map this signaling pathway was recently reported and it suggested the role of genes *RUNX1*, *RUNX3*, *SPINK4*, and *ELL2*.^163^

Other drugs that have strong effect on the IgG glycome are anti‐inflammatory biologicals like infliximab or vedolizumab.^211^, ^212^ Although there were some individuals that did not respond (and this group did not fully overlap with clinical non‐responders), in most people the introduction of anti‐inflammatory biologicals caused changes in the IgG glycome that were opposite of disease (and aging) effects. On the other hand, a number of drugs like statins and metformin that were found to associate with changes in the IgG glycome in cross‐sectional studies actually do not have any effect on IgG glycosylation.^213^, ^214^ Most probably this reflects effects of the underlying disease that is causally associated with changes in the IgG glycome and this confounding led to association between IgG glycome composition and drugs in cross‐sectional studies. In this respect, it is very important to properly control for potential confounders and when possible and perform placebo‐controlled studies to be able to distinguish real causal effects from associations caused by common confounders. For example, while there are numerous anecdotal indications that metformin has an anti‐aging effect, in the recent placebo‐controlled trial on healthy individuals only a single individual changed the IgG glycome into an anti‐inflammatory direction after introduction of metformin.^214^ However, in a diabetic population, over 50% of participants responded favorably to metformin. Similar rate of response was observed for SGLT2 inhibitors and GLP‐1 receptor agonists (unpublished data), suggesting that different molecular pathways may be responsible for the acceleration of glycan aging in different individuals.

## 10 PERSPECTIVES AND SPECULATIONS ON FUTURE DIRECTIONS IN IgG GLYCOSYLATION RESEARCH

Alternative glycosylation of IgG is an important regulatory mechanism of the immune system, but dynamics of IgG glycosylation and molecular mechanisms behind these changes are poorly understood. With notable exception of severe acute infections, when rapid changes in IgG glycome composition can be observed in a matter of days,^194^, ^196^ IgG glycome is generally very stable and changes only slowly with time. Acceleration of these changes are generally lifestyle and/or disease related, but causality of these relations is generally not known. Recent large study of individuals with the Down syndrome suggested that unrepaired DNA damage may be the underlying mechanism behind the accelerated glycans aging in the Down syndrome,^167^, ^215^ which is consistent with previous observations that DNA damage affects most, if not all, aspects of the aging phenotype.^216^ The observation that antiretroviral therapy also induces accelerated glycan aging is supportive of this hypothesis^217^, ^218^ as is the fact that in the Down syndrome the acceleration of glycan aging is restricted to very early stages of life, given that accelerated glycan aging has already been observed in childhood, while adults with Down syndrome age at the same pace as control individuals.^167^ However, this is probably just one of the mechanisms behind dynamics of the IgG glycome since a series of genome wide association studies (GWAS) identified over 40 genetic loci that regulate IgG glycosylation.^219^, ^220^, ^221^

Genetic network that regulates IgG glycosylation extends much further than just the enzymes that form the core glycosylation pathway. Enzymes appear to be simple “executors” or glycosylation, while the key “decision” about which glycans will be associated with different proteins is encoded somewhere else. Strong support for this hypothesis came from a recent study in which IgG and transferrin glycosylation was analyzed in parallel. While glycan structures attached to these to proteins are nearly all the same and the same enzymes create these structures, regulatory genes were found to be completely different.^222^ Interestingly, while there is some correlation between acceleration of glycan aging with different metabolomic or proteomic aging clocks, there is very little, or no correlation between acceleration of glycan aging and different epigenetic aging clocks.^223^ Recent study that performed glycosylation analysis of IgG secreted from different clones of B cells concluded that only Fc fucosylation is dominantly regulated at the single‐clone level, presumably through some heritable epigenetic mechanism, whereas the regulation of other glycosylation traits most likely occurs through external signaling.^224^

As GWAS is only a hypothesis generating tool, functional relevance for most of these genetic loci needs to be validated. The use of tools like EpiTools that enable direct manipulation of multiple candidate genes and subsequent analysis of effects on the IgG glycome^225^, ^226^, ^227^ are a promising approach, as it was recently demonstrated by mapping the downstream signaling mechanisms of oestradiol.^163^ However, in this type of studies one needs be careful to include proper controls, since IgG glycome secreted from cells can also be affected by the number of cell divisions in vitro.^228^

Genes that regulate IgG glycosylation are known risk factors for various diseases (autoimmune, inflammatory, cancer, etc),^219^, ^229^ which suggest that the impact of alternative glycosylation on the immune system might be mediating at least a part of disease pathology. IgG plays a crucial role in the immune system by activating inflammation through various mechanisms. However, its glycosylation status can shift its function to suppress inflammation. Although the precise molecular mechanisms remain unclear, it has been demonstrated that engineered sialylation of pathogenic antibodies in vivo can mitigate autoimmune diseases.^131^ Additionally, administering IVIg to patients with inflammatory diseases is a clinically established method to reduce inflammation, with this effect being dependent on glycosylation.^118^ Despite IVIg's effectiveness in suppressing inflammation, its use is limited by the fact that only a small fraction of IgG with appropriate glycosylation is active, and large quantities of human blood‐derived IgG are required. Ideally, developing a small‐molecule drug that could modulate host IgG glycosylation to render it anti‐inflammatory would be preferred.^230^ However, the mechanisms that regulate IgG glycosylation are still largely unknown, hindering the development of such therapies.

## 11 CONCLUSIONS

IgG glycans are an essential component of the IgG molecule that directly affect interactions between Fc receptors and other effector proteins. As we age, composition of the IgG glycome gradually shifts from predominantly galactosylated and sialylated glycans toward mostly agalactosylated glycans that dominate IgG glycome of old individuals. In inflammatory diseases (and other diseases that have an inflammatory component), this process is accelerated, and IgG glycome of people with the disease resembles glycome of much older individuals. Lifestyle interventions and some anti‐inflammatory drugs can revert this process and “rejuvenate” the IgG glycome, reflecting and/or promoting the decrease in chronic systemic inflammation. While mechanisms that regulate changes in glycosylation and molecular mechanisms behind extensive interindividual differences of IgG glycome composition still needs to be discovered, monitoring of individual's IgG glycome is a reliable proxy for different unfavorable health outcomes and has already been demonstrated as a useful tool for personalization of different lifestyle interventions.

## FUNDING INFORMATION

This work was funded by the European Union's Horizon Europe research and innovation program under the following Grant Agreements: ERC GlycanSwitch (Grant No. 101071386), Synhealth (Grant No. 101159018, synhealth‐project.eu), and geneTIGA (Grant No. 101057438, geneTIGA‐horizon.eu). Views and opinions expressed are, however, those of the author(s) only and do not necessarily reflect those of the European Union, the ERC Executive Agency, the European Research Executive Agency (REA), or the European Health and Digital Executive Agency (HADEA). Neither the European Union nor the granting authority can be held responsible for them.

## CONFLICT OF INTEREST STATEMENT

GL is founder and CEO and JK is employee of Genos, biotech company that is focusing on high‐throughput glycomics and has several patents in the field of glycan biomarkers. GL is a co‐founder and CSO of GlycanAge, biotech company that is developing and commercializing glycan biomarkers for biological age and disease prediction.

## Data Availability

Data are available upon request.
